# The inhibition of fibril formation of lysozyme by sucrose and trehalose†

**DOI:** 10.1039/d4ra01171f

**Published:** 2024-04-15

**Authors:** Kajsa Ahlgren, Fritjof Havemeister, Julia Andersson, Elin K. Esbjörner, Jan Swenson

**Affiliations:** a Division of Nano-Biophysics, Department of Physics, Chalmers University of Technology Gothenburg SE-412 96 Sweden kajsa.ahlgren@chalmers.se; b Division of Chemical Biology, Department of Life Sciences, Chalmers University of Technology Gothenburg SE-412 96 Sweden

## Abstract

The two disaccharides, trehalose and sucrose, have been compared in many studies due to their structural similarity. Both possess the ability to stabilise and reduce aggregation of proteins. Trehalose has also been shown to inhibit the formation of highly structured protein aggregates called amyloid fibrils. This study aims to compare how the thermal stability of the protein lysozyme at low pH (2.0 and 3.5) is affected by the presence of the two disaccharides. We also address the anti-aggregating properties of the disaccharides and their inhibitory effects on fibril formation. Differential scanning calorimetry confirms that the thermal stability of lysozyme is increased by the presence of trehalose or sucrose. The effect is slightly larger for sucrose. The inhibiting effects on protein aggregation are investigated using small-angle X-ray scattering which shows that the two-component system consisting of lysozyme and water (Lys/H_2_O) at pH 2.0 contains larger aggregates than the corresponding system at pH 3.5 as well as the sugar containing systems. In addition, the results show that the particle-to-particle distance in the sugar containing systems (Lys/Tre/H_2_O and Lys/Suc/H_2_O) at pH 2.0 is longer than at pH 3.5, suggesting larger protein aggregates in the former. Finally, the characteristic distance separating β-strands in amyloid fibrils is observed for the Lys/H_2_O system at pH 2.0, using wide-angle X-ray scattering, while it is not clearly observed for the sugar containing systems. This study further shows that the two disaccharides stabilise the native fold of lysozyme by increasing the denaturation temperature. However, other factors, such as a weakening of hydrophobic interactions and hydrogen bonding between proteins, might also play a role in their inhibitory effect on amyloid fibril formation.

## Abstract 2

WAXS displays a significant difference due to the presence of amyloid fibrils in the absence of sugar.

## Graphical abstract

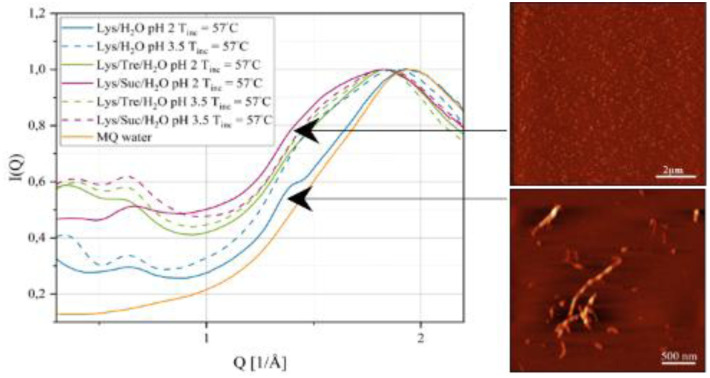

## 1 Introduction

Neurodegenerative diseases, such as Alzheimer's and Parkinson's, affect millions of people worldwide.^1,2^ They emerge when neurons in the brain gradually lose their function and begin to degenerate. Currently, there is no cure for these diseases, and only limited treatments to reduce symptoms.^2^ However, two new drugs, that slow the progression of Alzheimer's disease, were FDA-approved in 2023.^3^ Central in the development of improved treatment is to expand our knowledge of protein folding and misfolding, since neurodegenerative diseases are associated with protein aggregation. Amyloid fibrils are highly structured, linear protein aggregates. They are not only associated with neurodegenerative diseases but are implicated in a group of diseases known as amyloidoses.^4^

Hereditary lysozyme amyloidosis is an extremely rare condition^5^ in which the normally soluble protein lysozyme forms insoluble fibrillar structures due to abnormal folding.^6–9^ Deposits of these fibrillar structures in various tissues of the body gradually interfere with their normal structure and function.^5,10,11^ Lysozyme from hen egg white (HEWL) has been widely used as a model protein to study the formation of amyloid fibrils.^12,13^ It is a small single-chain protein consisting of 129 amino acids and is known to form amyloid fibrils at low pH and elevated temperatures.^12,14^ Amyloid fibrils, from HEWL, consist of two parallel protofilaments twisted around each other. These filaments, in turn, consist of HEWL monomers in cross-β conformation separated by 10–11 Å and stacked perpendicular to the axis of the fibril with a monomer-to-monomer separation of ∼4.7 Å. This gives rise to characteristic anisotropic patterns within amyloid fibrils which were first discovered using X-ray diffraction.^15,16^

Disaccharides are carbohydrates consisting of two monosaccharides linked together *via* a glycosidic linkage. It is well known that disaccharides possess a stabilising and anti-aggregating effect on proteins. This effect has been broadly studied at various conditions and for different proteins.^17–19^ The disaccharide trehalose has been of particular interest due to its superior ability to work as a cryoprotectant. In nature, trehalose is present in a wide range of extremophiles, such as the tardigrade,^20^ which can survive extreme conditions thanks to the presence of trehalose. The sugar has been used successfully as a cryoprotectant of membranes and vaccines as well as of plant and animal cells.^20^ Trehalose has been seen to disrupt the tetrahedral intermolecular network of water and form a glassy matrix to avoid ice formation.^21^ It has been suggested that this disruption is more pronounced for trehalose, compared to other disaccharides, due to the interaction between trehalose and water.^22^ Trehalose and the structurally similar disaccharide, sucrose, both have stabilising effects on proteins. However, trehalose has been seen to be superior with regard to cryopreservation. Despite the high interest in the subject, it is still unclear as to why disaccharides have a stabilising and anti-aggregating effect upon proteins.

In a previous study, we compared the structural and dynamical properties of trehalose and sucrose solutions and how they, together with water, interact with the protein myoglobin, using neutron scattering and molecular dynamics simulations.^23^ We found that, in both cases, myoglobin is hydrated by the surrounding water molecules and that the sugar molecules are preferentially excluded from the surface of the protein. The interaction between the protein surface and sucrose was higher compared to trehalose, although the interactions were quite small in both cases. The rotational motion around the dihedrals is faster for sucrose compared to trehalose. This, combined with the fact that sucrose was seen to bind slightly more to myoglobin compared to trehalose, possibly induces motions of the protein backbone, leading to destabilisation of the native state of the protein. In addition, the dynamics of the water was greatly reduced by the presence of trehalose which in turn reduced the dynamics of the protein.^24^ In contrast, the dynamics were not affected as greatly by the presence of sucrose.

Proteins need to be unfolded or partially unfolded to aggregate and form amyloid fibrils. The denaturation temperature (*T*_den_) is therefore a measure of the stability of a protein. Various studies suggest that trehalose is superior to sucrose in increasing *T*_den_ of proteins.^25–27^ However, Starciuc *et al.*^28^ found that *T*_den_ of lysozyme is higher with the addition of sucrose, compared to trehalose, in the presence of residual water. Moreover, James and McManus^29^ investigated the thermal stability of lysozyme from chicken egg white in different buffer conditions at different pH values and in the presence of either sucrose, glucose or trehalose. They found that both sucrose and glucose increase *T*_den_ slightly more than trehalose in all cases.

It has previously been shown that both sucrose and trehalose hinders the formation of amyloid fibrils of various proteins.^13,30^ In this study, we aim to compare the effect the two disaccharides have upon the formation of HEWL fibrils. We have studied and compared the intramolecular structures of HEWL fibrils as well as longer distances separating particles using WAXS and SAXS, respectively. The thermal denaturation of HEWL has been monitored using differential scanning calorimetry (DSC), which is a commonly used technique to study thermally induced events, such as denaturation and crystallisation, in various systems.^31,32^ To observe the effect of the disaccharides as well as presence of HEWL fibrils on the water structure the systems were analysed by the use of both WAXS and DSC. Finally, the presence of amyloid fibrils has been confirmed using atomic force microscopy (AFM).

## 2 Materials and methods

### 2.1 Sample preparation and fibril formation

d-(+)-Trehalose (dihydrate) and sucrose (anhydrous) (purchased from Sigma-Aldrich) were dissolved in MQ water under continuous stirring. The sugar : water weight ratio was 1 : 2 for both the Lys/Tre/H_2_O and Lys/Suc/H_2_O system. Thereafter, powdered lysozyme from hen egg white (HEWL) (purchased from Sigma Aldrich. Roche, product number: 10837059001) was added to the sugar solutions (in the Lys/H_2_O system it was dissolved in pure MQ water) without any further purification. Due to the high protein concentration, HEWL was dissolved little by little under gentle stirring. For the Lys/H_2_O system the protein : water ratio was kept equal to that of the sugar-containing systems. The pH of the systems was, without adjustment, ∼3.5. HEWL has been seen to form fibrils at pH 2.0. The pH was, therefore, adjusted to 2.0 using 2 M HCl and was measured using both a Mettler Toledo Seven2go pH-meter and pH sticks. The systems at pH 3.5 were used as reference systems. All prepared systems are listed in Table 1. Six different systems were prepared, Lys/H_2_O, Lys/Tre/H_2_O, and Lys/Suc/H_2_O at pH 2.0 and 3.5, all incubated at *T*_inc_ = 57 °C according to literature.^33–36^ This was done to partly unfold the protein, which is required for the formation of amyloid fibrils. Four additional systems were prepared, Lys/Tre/H_2_O, and Lys/Suc/H_2_O at pH 2.0 and 3.5, now incubated at *T*_inc_ = 65 °C and *T*_inc_ = 68 °C, respectively. The incubation temperatures for the sugar containing systems were increased in order to exclude the explanation that the inhibition of amyloid fibrils was solely due to an elevated denaturation temperature caused by the presence of disaccharides. The temperatures were chosen based on how much the denaturation temperature was increased for each system. All systems were incubated for 24 h.

**Table 1:** The six different systems (with or without sugar) with an incubation temperature of *T*_inc_ = 57 °C (shown as normal values) and the four additional, sugar containing, systems with an incubation temperature of *T*_inc_ = 65 °C and *T*_inc_ = 68 °C for trehalose ans sucrose respectively (shown within parentheses)

System	Conc. [wt%]	*T*_inc_ [°C]
**pH 2.0**		
Lys/H_2_O	27/73	57
Lys/Tre/H_2_O	20/27/53	57 (65)
Lys/Suc/H_2_O	20/27/53	57 (68)

**pH 3.5**		
Lys/H_2_O	27/73	57
Lys/Tre/H_2_O	20/27/53	57 (65)
Lys/Suc/H_2_O	20/27/53	57 (68)

### 2.2 X-ray scattering

Two commonly used techniques to probe the structure of materials and solutions on a nanometre scale are small- and wide-angle X-ray scattering (SAXS/WAXS). In this work, SAXS and WAXS have been used to study the inter- and intra-molecular distances within the systems, respectively. The two techniques operate on the same principle, where the placement of the detector is the main difference. All SAXS and WAXS experiments were performed in vacuum at room temperature at Chalmers Material Analysis Laboratory (CMAL), using a Mat:Nordic instrument (SAXSLAB). The samples were injected into quartz capillary tubes, with a thickness of 1.5 mm, using a syringe. The exposure time of each sample was 5 and 15 min for WAXS and SAXS, respectively. In SAXS, the detector was positioned 1081 mm away from the sample, corresponding to a *Q* range of 0.00138–0.30717 Å^−1^, which enabled analysis of greater distances between particles in the system. As a complement, WAXS enables the analysis of smaller distances by having the detector positioned 131 mm from the sample, corresponding to a *Q* range of 0.00381–2.2397 Å^−1^. The obtained peaks correspond to the distance between the scattering centres and are present due to characteristic distances within the sample. Each recorded peak was fitted by a Gaussian to obtain the peak position at maximum. Finally, the real-space centre-to-centre distance was calculated from the peak position at maximum using *d* = 2π/*Q*, where *Q* is the length of the scattering vector and *d* is the distance in real-space. SAXS/WAXS are commonly used to gain information regarding the structure and arrangement within biological systems.^37,38^

### 2.3 Differential scanning calorimetry

DSC experiments were performed to monitor both the crystallisation temperature (*T*_c_) of water and the thermally induced denaturation of HEWL. The DSC measurements were performed on a DSC Q2000 (TA Instruments). For studies of the denaturation, the samples were heated from 5 °C to 90 °C with a heating rate of 2 °C min^−1^. Subsequently, they were cooled from 90 °C to 5 °C at a cooling rate of 10 °C min^−1^. Cycles of three were executed to examine reversibility of the denaturation step and examine whether other features occur due to thermal history. The dependence of the protein concentration was measured on samples with no prior incubation or altering of the pH. The samples were measured in an aluminium hermetic pan. *T*_den_ was determined by locating the midpoint of the characteristic endothermal peak caused due to an increase in entropy.

The crystallisation temperature (*T*_c_) was measured by cooling from 20 °C to −60 °C with a cooling rate of 10 °C min^−1^ followed by a heating ramp from −60 °C to 40 °C at a rate of 10 °C min^−1^. The crystallisation event is portrayed as a dramatic exothermal peak due to the decrease in entropy. *T*_c_ was determined from the onset of the peak and the change in enthalpy, Δ*H*, was determined by integrating the endothermal melting peak.

### 2.4 Atomic force microscopy

To further confirm the presence of possible fibrils AFM was used. All samples were diluted 1 : 20 in order to observe individual structures more clearly, except for the Lys/H_2_O system at pH 2.0 which was diluted 1 : 40. The diluted samples were pipetted onto mica plates and allowed to settle for 10 min in room temperature. The plates were then rinsed 10 times with MQ water, and carefully dried with filter paper and nitrogen. AFM images were recorded on an NT-MDT NTEGRA Prima atomic force microscope with a NSG01 gold-coated single crystal silicon probe (resonant frequency ∼150 kHz, force constant ∼5.1 N m^−1^) using tapping mode (0.5 Hz scan frequency). The AFM imaging was performed in air. The images were processed using Gwyddion.^39^

## 3 Results and discussion

### 3.1 Anti-aggregation and inhibition of fibril formation

**Fig. 1:**
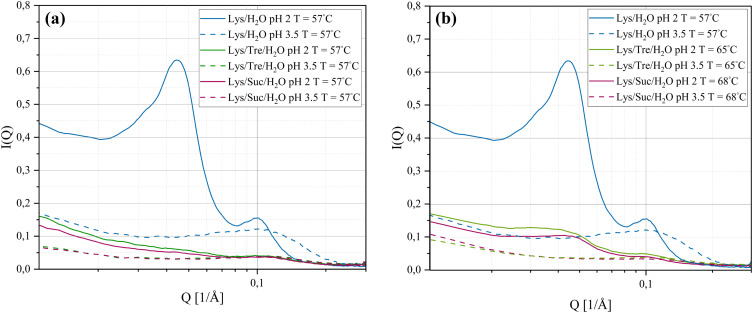
SAXS data of (a) Lys/H_2_O, Lys/Tre/H_2_O, and Lys/Suc/H_2_O systems at pH 2.0 and pH 3.5, *T*_inc_ = 57 °C and (b) Lys/Tre/H_2_O, and Lys/Suc/H_2_O systems at pH 2.0 and pH 3.5, *T*_inc_ = 65 °C respectively 68 °C (the two Lys/H_2_O systems are added for reference, however, is the same data as in (a)).

SAXS was used to investigate intermolecular distances ranging from tens to hundreds of Ångströms. Understanding the distances at this scale offers insights into the spatial distribution of molecules in the sample. The SAXS curves for the systems listed in Table 1 (incubated at 57 °C for 24 h) are displayed in Fig. 1(a). For the Lys/H_2_O system at pH 2.0, two distinct peaks are evident. Notably, the peak appearing in the lower *Q*-range (*Q*_1_ = 0.0447 Å^−1^, *d*_1_ = 141 Å^−1^) is absent in the other five systems. Peaks in the lower *Q*-range correspond to greater distances in real-space, whereas peaks in the higher *Q*-range indicate shorter distances. The greater distances most likely arise from more aggregation of the protein, such as the formation of fibrillar structures or amorphous aggregates. Based on the data in Fig. 1(a), it can be concluded that the Lys/H_2_O system at pH 2.0 exhibits two well-defined distances, suggesting a bimodal size distribution. In contrast, the other five systems display only one weak and broad peak due to a characteristic but not well defined protein-to-protein distance. It is known from literature that HEWL forms amyloid fibrils at pH 2.0 after incubation at elevated temperatures.^12,14,33,40^Fig. 1 suggests a significant structural difference between the Lys/H_2_O system at pH 2.0 and 3.5. The structural difference that is seen for the two-component system can also be observed for the systems with trehalose or sucrose, but the differences are smaller in these cases. The SAXS data suggest that, despite a pH of 2.0 and incubation at 57 °C for 24 h, the disaccharides are able to inhibit the formation of larger aggregates. These results indicate that the two disaccharides inhibit the aggregation of HEWL, despite harsh pH conditions.

Fig. 1(b) displays the Lys/Tre/H_2_O system incubated at 65 °C for 24 h and the Lys/Suc/H_2_O system incubated at 68 °C for 24 h.‡‡The Lys/H_2_O systems are identical to those shown in Fig. 1(a). When *T*_inc_ for the three-component systems were increased closer to the observed respective denaturation midpoints, which were revealed using DSC as further described below, an indication of a peak in the lower *Q*-range appeared also for the Lys/Tre/H_2_O and Lys/Suc/H_2_O systems at pH 2.0. For Lys/Tre/H_2_O, the peak appears at *Q*_1_ = 0.0417 Å^−1^, corresponding to a real-space distance of *d*_1_ = 151 Å, whereas for Lys/Suc/H_2_O it appears at *Q*_1_ = 0.0457 Å^−1^, translating to *d*_1_ = 138 Å. Since the three-component systems are more crowded compared to the two-component systems it is difficult to compare these numbers on the basis of the amount of aggregation. However, the peaks in the three-component systems are less pronounced, indicating a less distinct repetition of the distance. A comparison of the Lys/Tre/H_2_O and Lys/Suc/H_2_O systems at the two pH values and at different *T*_inc_ can be found in Fig. S1 in the ESI.†

**Fig. 2:**
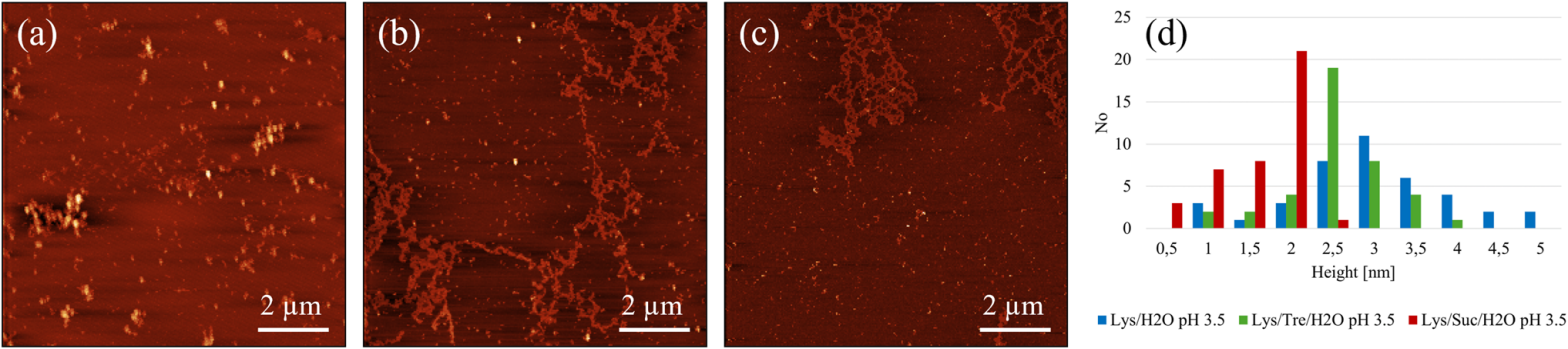
Representative AFM images of (a) Lys/H_2_O, (b) Lys/Tre/H_2_O, and (c) Lys/Suc/H_2_O at pH 3.5. (d) Shows an analysis of the height of the structures, where the blue, green, and red poles represent the height of the fibrillar structures in Lys/H_2_O, Lys/Tre/H_2_O and Lys/Suc/H_2_O at pH 3.5, respectively. The AFM imaging was performed in air.

**Fig. 3:**
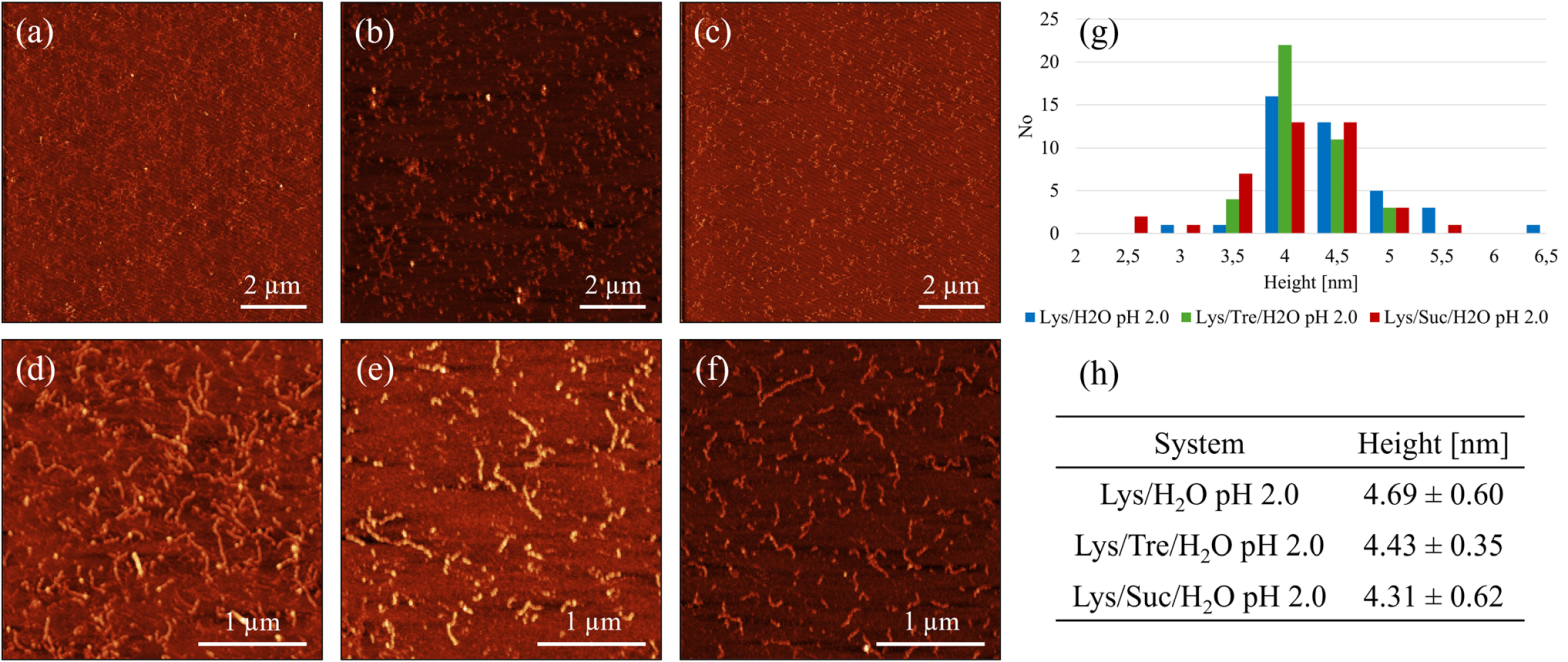
Representative AFM images of (a) Lys/H_2_O, (b) Lys/Tre/H_2_O, and (c) Lys/Suc/H_2_O at pH 2.0. (d), (e) and (f) show magnified scans of (a), (b) and (c), respectively. (g) Shows an analysis of the height of the structures, where the blue, green, and red poles represent the height of the fibrillar structures in Lys/H_2_O, Lys/Tre/H_2_O and Lys/Suc/H_2_O, respectively. (h) Shows the average heights. The AFM imaging was performed in air.

A more detailed analysis of the peak positions and their corresponding real-space distances is provided in Table 2. The peak in the higher *Q*-range occurs at a *Q*-value of 0.103 Å^−1^, translating to a real-space distance of 61.0 Å for the Lys/H_2_O system at pH 2.0. As previously noted, the Lys/H_2_O system at pH 3.5 displays a peak solely in the higher *Q*-range, at a *Q*-value of 0.113 Å^−1^ (55.6 Å in real-space). In contrast to the more acidic system, this peak is broader indicating a higher level of polydispersity. Assuming a system consisting of evenly distributed protein molecules, the centre-to-centre separation would be approximately 43.8 Å. A system containing larger protein aggregates would be less crowded, hence more spacious, compared to a system containing many protein monomers. It is therefore suggested that aggregation occurs in both systems. However, larger structures are only present in the system at pH 2.0 whereas the aggregates in the system at pH 3.5 seem to be more heterogeneous. Fig. 2(a) shows an AFM image of the Lys/H_2_O system at pH 3.5. Comparing this system with the two-component system at pH 2.0 (see Fig. 3(a) and (d)), no amyloid fibrils can be observed in the less acidic system. An analysis of the height of the aggregates in the Lys/H_2_O system at pH 3.5 is presented as blue poles in the histogram shown in Fig. 2(d). The AFM image of Lys/H_2_O at pH 3.5 including a colour map of the height of the aggregates can be seen in Fig. S3 in the ESI.† In addition, the larger amorphous aggregate networks observed for the sugar containing systems at pH 3.5 (see Fig. 2(b) and (c)) was not observed for Lys/H_2_O at pH 3.5 which can explain why the protein-to-protein distance is longer for the sugar containing systems at pH 3.5 compared to at pH 2.0. Arnaudov and de Vries^33^ studied the morphology of fibrillar aggregates of lysozyme at pH 2, 3, and 4 at different temperatures and found that fibrils were formed at pH 3, *T*_inc_ = 57 °C only after 11 days of incubation. However, it is worth mentioning the difference in protein concentration, where our samples contain ∼20 times more protein compared to theirs.

**Table 2:** Peak positions, recorded by SAXS, where the normal values are for the systems incubated at 57 °C and the values within parentheses are for the Lys/Tre/H_2_O and Lys/Suc/H_2_O systems incubated at *T* = 65 °C and *T* = 68 °C, respectively. *x* indicates the lack of peak

System	*Q*_1_ [Å^−1^]	*d*_1_ [Å]	*Q*_2_ [Å^−1^]	*d*_2_ [Å]
**pH 2.0**				
Lys/H_2_O	0.0447	141	0.103	61.0
Lys/Tre/H_2_O	*x* (0.042)	*x* (151)	0.119 (0.100)	52.9 (62.8)
Lys/Suc/H_2_O	*x* (0.046)	*x* (138)	0.120 (0.106)	52.4 (59.3)

**pH 3.5**				
Lys/H_2_O	*x*	*x*	0.113	55.6
Lys/Tre/H_2_O	*x* (*x*)	*x* (*x*)	0.113 (0.112)	55.6 (56.1)
Lys/Suc/H_2_O	*x* (*x*)	*x* (*x*)	0.115 (0.115)	54.6 (54.9)

In the Lys/Tre/H_2_O system at pH 2.0 (*T*_inc_ = 57 °C) a peak at *Q*_2_ = 0.119 Å^−1^ (*d*_2_ = 52.9 Å) is present, while at pH 3.5 the peak is located at a slightly lower *Q*-value of 0.113 Å^−1^ (*d*_2_ = 55.6 Å). This shift suggests that the protein-to-protein distance is shorter in the more acidic system. In Fig. 2(b) an AFM image of the Lys/Tre/H_2_O system at pH 3.5 is presented in which large amorphous aggregate networks can be observed. These large unstructured aggregates can explain the longer protein-to-protein distance compared to the Lys/Tre/H_2_O system at pH 2.0. The heights of these structures were analysed, and are presented as green poles in the histogram shown in Fig. 2(d). Amyloid fibrils formed from HEWL usually measure a height of approximately 4.0 nm.^33^ The average height of the structures observed in Fig. 2(b) is approximately 2.8 nm, which combined with a visual inspection indicates that the observed aggregates are not fibrillar structures. An AFM image of the Lys/Tre/H_2_O system at pH 3.5 including a colour map of the height distribution can be seen in Fig. S4 in the ESI.† The Lys/Suc/H_2_O system at pH 2.0 and pH 3.5 (*T*_inc_ = 57 °C) displays peaks at *Q*-values of 0.120 Å^−1^ (*d*_2_ = 52.4 Å) and 0.115 Å^−1^ (*d*_2_ = 54.6 Å), respectively. Similarly to the systems with trehalose, the acidic system displays a shorter protein-to-protein distance, which can be explained by observing the larger amorphous aggregates in the Lys/Suc/H_2_O system at pH 3.5 (see Fig. 2(c). The average height of the observed aggregates in the Lys/Suc/H_2_O system is approximately 1.9 nm, which indicates that the observed structures are not amyloid fibrils. An AFM image of the Lys/Suc/H_2_O system at pH 3.5 including a colour map of the height distribution can be seen in Fig. S5 in the ESI.† The large areas of amorphous aggregates are consistent with the polydispersity observed in SAXS. In a system with either disaccharide, the average centre-to-centre separation in a homogeneously distributed sample would be 47.4 Å. Considering the protein-to-protein distance in the three-component systems in this study being longer than 47.4 Å, some kind of aggregation is suggested to occur also in all sugar-containing systems. Lastly, it is important to highlight that weak broad peaks are observed across all three-component systems, which indicates a high level of polydispersity.

Even though SAXS shows that a higher level of aggregation is occurring in the Lys/H_2_O system at pH 2.0 compared to the sugar containing systems at the same pH, it does not confirm that these protein aggregates are amyloid fibrils. However, from the AFM images displayed in Fig. 3, it could be seen that mature amyloid fibrils are present in the Lys/H_2_O system at pH 2.0. Fig. 3(a) shows amyloid fibrils homogeneously distributed over the surface of a mica plate. In Fig. 3(d) a magnified image of Fig. 3(a) can be seen. The fibrils in this system appears to be 3–7 nm high and 100 nm to 1.5 μm long. As previously mentioned, mature amyloid fibrils formed from HEWL typically have a height of ∼4.0 nm,^33^ whereas protofibrils are typically lower. The analysis of the average height of the fibrillar structures observed in the Lys/H_2_O system at pH 2.0 suggests that both protofibrils and mature amyloid fibrils are present. An alternative image of an amyloid fibril, displaying the characteristic amyloid twist along the axis of the fibril, in the Lys/H_2_O system at pH 2.0 can be seen in Fig. S6 in the ESI.†Fig. 3(b) shows the presence of small aggregates in the Lys/Tre/H_2_O system at pH 2.0, which could be of amyloid type. A magnified AFM image can be seen in Fig. 3(e), displaying the presence of fibrillar structures. The height of the aggregates observed in the trehalose system at pH 2.0 is 3.5–5 nm (see Fig. 3(g)) and the average is presented in Fig. 3(h). The height of the observed aggregates suggests that both protofibrils and mature amyloid fibrils are present in this system. However, the SAXS data suggest that the concentration of such aggregates is much lower compared to the Lys/H_2_O system at pH 2.0. This is also in agreement with the SAXS data showing a protein-to-protein separation only slightly longer than the distance between homogeneously distributed monomers. An AFM image of the Lys/Suc/H_2_O system at pH 2.0 is presented in Fig. 3(c), suggesting the formation of similarly small aggregates as in the trehalose system. Fig. 3(f) shows a magnified AFM image of the Lys/Suc/H_2_O system at pH 2.0. The height of the observed aggregates is 2.5–5.5 nm (see Fig. 3(g)) and the average height is presented in Fig. 3(h). Similarly to the Lys/Tre/H_2_O system at pH 2.0, the height indicates that the observed structures are both protofibrils and mature amyloid fibrils. However, the absence of a peak in the lower *Q*-range of the SAXS data indicates that the concentration of fibrillar structures in the Lys/Suc/H_2_O system at pH 2.0 is much lower compared to that of the two-component system. The AFM images of the systems at pH 2.0 including a colour map of the height distribution can be seen in Fig. S7, S8, and S9 in the ESI.†

**Fig. 4:**
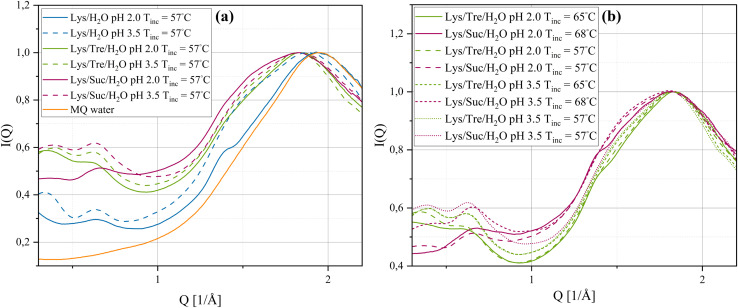
Normalised WAXS data of (a) Lys/H_2_O, Lys/Tre/H_2_O, and Lys/Suc/H_2_O systems at pH 2.0 and pH 3.5, all systems were incubated at 57 °C. (b) The three-component systems at pH 2.0 and pH 3.5 incubated at either 57 °C or 68 °C for Lys/Suc/H_2_O and at either 57 °C or 65 °C for Lys/Tre/H_2_O. The orange solid line in (a) shows the WAXS data of pure MQ water.

By the use of WAXS, distances on a shorter length scale, such as those within fibrils or between water molecules can be probed. Fig. 4(a) shows, the normalised§§The curves are normalised against the peak maximum of the water-structure. WAXS data of the systems incubated at 57 °C. The orange solid line represent the normalised WAXS data of pure MQ water. The Lys/H_2_O system at pH 2.0 shows an indication of a smaller peak, displayed as a “shoulder” to the left of the peak corresponding to the distance separating two water molecules. This shoulder peak appears around *Q* = 1.38 Å^−1^, which corresponds to a real-space distance of *d* = 4.55 Å. This distance is reasonably close to the typical interstrand distance of amyloid fibrils, suggesting the presence of such aggregates. For the Lys/H_2_O system at pH 3.5 and all sugar containing systems at either pH 2.0 or pH 3.5 this peak was not found or was too weak to be identified. For a high concentration of mature fibrils the interstrand distance is repeated multiple times and should therefore give rise to a much more pronounced peak.^16^ This suggests that mature fibrils are not formed after incubation at 57 °C for 24 h in the sugar-containing systems. In addition, all systems have an additional peak in the *Q*-range of 0.639–0.677 Å^−1^. A peak in this *Q*-range has previously been proposed, by Hirai *et al.*,^41,42^ to correspond to the distance separating two alpha-helices in the secondary structure of lysozyme. By examining the intensity of the peak at this *Q*-range it is seen to decrease for the systems at pH 2.0. This indicates a reduction of such alpha-helices in these systems which would be expected upon HEWL denaturation. No considerable difference in the peak intensity, of the peak in the *Q*-range of 0.639–0.677 Å^−1^, could be seen between the Lys/Tre/H_2_O and Lys/Suc/H_2_O systems. It should be mentioned that the distance associated with the intersheet distance of amyloid fibrils would also give rise to a peak at a similar *Q*-range. However, considering the amount of free monomers still left in the solution after the short incubation time of 24 h also in the Lys/H_2_O system at pH 2.0, it is expected that the distance separating two alpha-helices in the native fold of HEWL occurs more frequently compared to the intersheet distance of amyloid fibrils. McAllister *et al.*^43^ studied the conformational changes of HEWL at different pH values ranging from pH 1.0–9.8 using circular dichroism. They observed a slight decrease of the alpha-helical content by decreasing the pH from 3.8 to 2.0, which is consistent with what we observe here.

In Fig. 4(b) the WAXS data of the Lys/Tre/H_2_O and Lys/Suc/H_2_O systems incubated at *T*_inc_ = 65 °C and *T*_inc_ = 68 °C, respectively are displayed. The position of the peak associated with the water structure remains unchanged. However, the small bump, indicated to the left of the water structure peak at *T*_inc_ = 57 °C, is more pronounced for both the Lys/Tre/H_2_O and Lys/Suc/H_2_O systems at pH 2.0 at their new respective *T*_inc_, indicating more fibrillar structures in these systems compared to the corresponding systems incubated at *T*_inc_ = 57 °C. This was confirmed by AFM and can be seen in Fig. S10 in the ESI.† The peak positions and their relative real-space distances are shown in Table 3.

**Table 3:** The *Q*-value of maximum intensity recorded by WAXS. The normal values are for the systems incubated at *T*_inc_ = 57 °C while the values within parenthesis are for the Lys/Tre/H_2_O and Lys/Suc/H_2_O systems incubated at *T*_inc_ = 65 °C and *T*_inc_ = 68 °C, respectively. *x* indicates the lack of peak

System	*Q*_1_ [Å^−1^]	*d*_1_ [Å]	*Q*_2_ [Å^−1^]	*d*_2_ [Å]	*Q*_3_ [Å^−1^]	*d*_3_ [Å]
**pH 2.0**						
Lys/H_2_O	0.639	9.83	1.38	4.55	1.95	3.22
Lys/Tre/H_2_O	0.644 (0.633)	9.76 (9.93)	*x* (1.41)	*x* (4.46)	1.83 (1.85)	3.43 (3.39)
Lys/Suc/H_2_O	0.677 (0.647)	9.28 (9.71)	*x* (1.38)	*x* (4.55)	1.83 (1.83)	3.43 (3.43)

**pH 3.5**						
Lys/H_2_O	0.640	9.82	*x*	*x*	1.88	3.34
Lys/Tre/H_2_O	0.650 (0.644)	9.67 (9.75)	*x* (1.47)	*x* (4.27)	1.83 (1.84)	3.43 (3.41)
Lys/Suc/H_2_O	0.644 (0.667)	9.76 (9.42)	*x* (1.46)	*x* (4.30)	1.85 (1.84)	3.40 (3.41)

From the WAXS data it can be seen that all systems exhibit clear peaks (*Q*_3_) corresponding to the distance between two water molecules. When compared to the peak recorded for pure MQ water there is a shift to lower *Q*-values for all systems, with the exception of the Lys/H_2_O system at pH 2.0. This indicates that the water molecules in this system are arranged similar to those in bulk water, which is reasonable considering there are larger protein aggregates present in this system therefore also more bulk-like water. It has been seen in previous studies that disaccharides disrupt the tetrahedral network in amorphous water.^44–46^ This can also be seen in Fig. S2 in the ESI,† where the sugar solutions are compared with pure MQ water. In a system with larger protein aggregates, the water in the system would behave more bulk-like compared to a system where the protein molecules are homogeneously distributed since the amount of hydration water is less in the former. One way to examine the homogeneity of a sample and the area of protein surface which amorphous hydration water can interact with is to study the fraction of the water that does not crystallise at any temperature. If the water in a sample exhibits a *T*_c_ and Δ*H* value close to that of pure MQ water (−17.1 °C and 333.1 J g^−1^, respectively) the presence of such amorphous hydration water is low. In Table 4, *T*_c_, Δ*H* (normalised to the amount of water in the sample), and the percent amorphous water (Am H_2_O) in the sample of all systems at pH 2.0 and 3.5 (*T*_inc_ = 57 °C) are presented. For the Lys/H_2_O system at pH 2.0 it can be seen that the Δ*H* is slightly higher compared to at pH 3.5, suggesting regions of more bulk-like water which is most likely due to the formation of larger aggregates in the former. The fraction amorphous water that does not crystallise is less in the system at pH 2.0 than at pH 3.5. This is in line with the results that the system at pH 2.0 contains larger aggregates compared to at pH 3.5 since the total surface area of the protein is smaller for larger aggregates, hence the amount of hydration water is less. Comparing the Lys/Tre/H_2_O and Lys/Suc/H_2_O systems the Δ*H* is slightly higher for the trehalose containing system at both pH values. In addition, the amount amorphous water that does not crystallise is less for the trehalose system, suggesting slightly larger aggregates in the trehalose systems which is in line with the SAXS data. Comparing the Δ*H* of the Lys/Tre/H_2_O system at pH 2.0 with that at pH 3.5 the difference is very small. A similar observation can be made for the Lys/Suc/H_2_O system, suggesting that the smaller protein aggregates observed in the sugar containing systems at pH 2.0 do not reduce the amount of hydration water more than the amorphous aggregates observed at pH 3.5. Representative DSC curves of the crystallisation event for systems Lys/H_2_O, Lys/Tre/H_2_O, and Lys/Suc/H_2_O at pH 2.0 can be seen in Fig. S11(a) of the ESI.† Additionally, respective curves for the systems at pH 3.5 can be seen in Fig. S11(b) of the ESI.† Since the disaccharides perturb the water structure it is expected that *T*_c_ is lower for the sugar containing systems. When HEWL forms fibrils a transparent gel can be observed. This gel formation could be a possible explanation to a lower *T*_c_ at pH 2.0 compared to the corresponding system at pH 3.5.

**Table 4:** The average crystallisation temperature, *T*_c_, the enthalpy change, Δ*H*, per mass H_2_O as well as the percentage amorphous H_2_O (Am H_2_O) in systems Lys/H_2_O, Lys/Tre/H_2_O, and Lys/Suc/H_2_O at pH 2.0 and 3.5 incubated at *T* = 57 °C

System	*T*_c_ [°C]	Δ*H* [J g^−1^ H_2_O]	Am H_2_O [%]
**pH 2.0**			
Lys/H_2_O	−19.4 ± 3.47	288 ± 8.77	13.6
Lys/Tre/H_2_O	−24.0 ± 1.25	237 ± 7.21	29.0
Lys/Suc/H_2_O	−29.5 ± 0.88	195 ± 2.12	41.4

**pH 3.5**			
Lys/H_2_O	−16.7 ± 4.15	228 ± 14.4	31.6
Lys/Tre/H_2_O	−25.2 ± 1.86	221 ± 5.25	33.6
Lys/Suc/H_2_O	−23.6 ± 5.86	200 ± 12.6	40.1

### 3.2 Increased thermal stability of HEWL

**Fig. 5:**
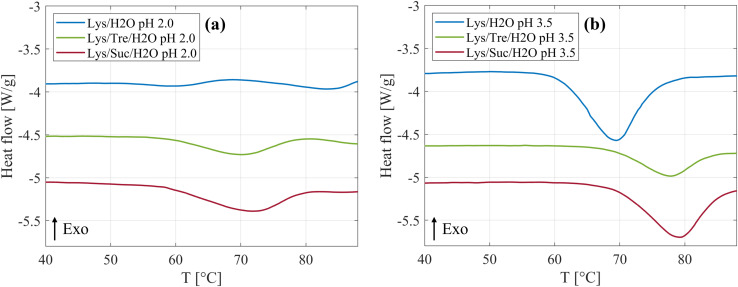
Representative DSC curves for the Lys/H_2_O, Lys/Tre/H_2_O, and Lys/Suc/H_2_O systems at (a) pH 2.0 and at (b) pH 3.5. All systems here were incubated at 57 °C. The curves are vertically shifted for clarity.

An increased thermal stability of proteins by addition of disaccharides has previously been observed in literature,^25–29^ and was also observed in this study. Fig. 5(a) shows the DSC curves of the systems at pH 2.0 incubated at 57 °C are displayed. It can be clearly seen that both disaccharides increase *T*_den_ of HEWL. In Table 5 a more detailed presentation of *T*_den_ can be seen. Compared to the Lys/H_2_O system at pH 2.0, *T*_den_ was increased by 12.6 °C and 9.5 °C for the Lys/Suc/H_2_O and Lys/Tre/H_2_O system, respectively. Thus, for these systems we report a slightly higher *T*_den_ for the system with sucrose compared to the system with trehalose. James and McManus^29^ investigated the thermal stability of lysozyme in the presence of sugars at different buffer conditions, similarly they found that sucrose increases *T*_den_ more than trehalose. However, there are many examples in literature suggesting that trehalose is superior in regards of increasing *T*_den_ of lysozyme.^25–27^ The samples have been incubated at 57 °C, a temperature slightly below *T*_den_. This condition not only influences the equilibrium between folded and unfolded monomers but also introduces the possibility that unfolded proteins could aggregate. Such aggregation could significantly alter the concentration of free monomers available in the solution. Consequently, the HEWL concentration dependency on *T*_den_ was investigated and can be seen in Fig. S12 in the ESI.† Additional endothermal peaks can be seen for all three systems above 80 °C. The same phenomenon was observed by Catalini *et al.*^47^ when highly concentrated lysozyme (18 wt%) dissolved in D_2_O was studied using micro-DSC. These peaks were suggested to be due to an entropy driven event or additional conformational changes of partly unfolded protein. In Fig. S13–S18 in the ESI,† the reversibility of the protein denaturation for the different systems can be seen.

**Table 5:** The mean denaturation temperature, *T*_den_, for the Lys/H_2_O, Lys/Tre/H_2_O, and Lys/Suc/H_2_O systems at pH 2.0 and 3.5 incubated at *T* = 57 °C for 24 h

System	*T*_den_ [°C]
**pH 2.0**	
Lys/H_2_O	58.3 ± 2.26
Lys/Tre/H_2_O	67.8 ± 2.53
Lys/Suc/H_2_O	70.9 ± 1.51

**pH 3.5**	
Lys/H_2_O	69.1 ± 0.37
Lys/Tre/H_2_O	76.8 ± 1.67
Lys/Suc/H_2_O	78.9 ± 0.33

The DSC curves for the systems at pH 3.5, *T*_inc_ = 57 °C, are displayed in Fig. 5(b). By comparing Fig. 5(a) and (b), it is clear that *T*_den_ is affected by altering the pH value, whether the disaccharides are present or not. Beg *et al.*^48^ investigated how heat induced denaturation is influenced by the pH of the system. They also saw that for lysozyme *T*_den_ decreased with decreasing pH, which is in line with what we present here. The isoelectric point (pI) of lysozyme is between 10.3–11 (ref. 49) which means that the protein at this pH value has a net charge of zero. At a more acidic pH it is therefore reasonable to assume that the protein would be less stable due to an increased protonation of acidic residues and a net positive charge leading to more intramolecular repulsion. In Table 5 it can be seen that sucrose increases *T*_den_ by 9.8 °C, whereas trehalose increases it by 7.7 °C at pH 3.5. Thus, it is clear that sucrose, also at this pH, increases *T*_den_ slightly more than trehalose. These data support the idea that the two disaccharides inhibit fibril formation by increasing *T*_den_. Poddar *et al.*^50^ found that the mixture glucose:fructose with a 1 : 1 ratio increases the denaturation temperature slightly more than pure sucrose. Seeing as sucrose is hydrolysed by HCl, and hence reduced into the two monosaccharides, this could be a possible explanation as to why sucrose in this case increases the denaturation temperature slightly more than trehalose. The hydrolysis of sucrose can be seen as a slight decrease of the intensity in the lower *Q*-range in the WAXS data in Fig. 4(b). In addition, it was suggested by Wang *et al.*,^51^ that sugars hinder fibril formation by stabilisation of the native state of protein, thus, hindering unfolding and subsequent fibrillation. However, it is still unclear weather this is the sole explanation. This was tested by increasing *T*_inc_ to 65 °C and 68 °C for the trehalose and sucrose containing systems, respectively, to make *T*_inc_ equally close to *T*_den_ as for the samples without any sugar. From WAXS the presence of a small shoulder in the *Q* range of 1.4–1.5 Å^−1^ could be observed for these systems, corresponding to the characteristic distance of 4.7 Å separating two β strands within an amyloid fibril. In addition, a peak in the lower *Q* range was observed using SAXS as *T*_inc_ was increased indicating the presence of larger aggregates. However, it should be noted that this peak is not as well pronounced as the peak observed in the same *Q*-range for the Lys/H_2_O system at pH 2.0 (see Fig. 1(b)) suggesting a less well defined distance. This is in agreement with the AFM images displaying small protein aggregates for the sugar containing systems after incubation at a higher temperature and mature amyloid fibrils for the Lys/H_2_O system at pH 2.0, see Fig. S10 in the ESI.† These results suggest that stabilisation of the native fold might not be the sole explanation to the inhibitory effect of the disaccharides. Liu *et al.*^52^ performed all-atom molecular dynamics simulations to study the inhibitory effect of trehalose on the nucleation and elongation of amyloid β-peptide oligomers and observed that the monomers were stabilised in the turn, bend, or coil, so that the β-rich structure was prevented. Additionally, they found that the preferential hydration of the protein together with the trehalose molecules weaken hydrophobic interactions between peptides and that the direct and indirect hydrogen bonds between the protein and trehalose suppress the hydrogen bonding between peptides. This could be a possible explanation also for the inhibitory effect of trehalose and sucrose on the formation of amyloid fibrils from HEWL.

In a previous study, regarding the stabilising effect of trehalose and sucrose on myoglobin, we could, through neutron diffraction, conclude that sucrose seems more prone to bind directly to the surface of the protein compared to trehalose, which exhibits a stronger preferential exclusion effect.^23^ This result provided information that trehalose is able to stabilise the native state of myoglobin with little to no direct interaction with the surface of the protein. In the same study we performed quasielastic neutron scattering in combination with molecular dynamics simulations and observed that the dynamics of the water in the system is slowed down by the presence of both trehalose and sucrose. However, the effect was more pronounced for the former. Rotational free energy calculations showed that the rotation around the dihedrals of sucrose is faster than that of trehalose. This together with the fact that sucrose binds more directly to the protein should be a disadvantage for sucrose as a stabilising agent. However, the finding that sucrose interacts slightly more with the surface of myoglobin might be beneficial in the case of inhibiting amyloid fibril formation of other proteins. The direct binding to the surface of the protein could restrict the conformational flexibility necessary for the formation of amyloid fibrils. Furthermore, the direct interaction may create a barrier, which prevents the exposure of hydrophobic, aggregation prone regions. Thus, the stabilising effect of the two disaccharides observed in the case with myoglobin is expressed slightly differently for lysozyme, which gives us reason to believe that the two disaccharides may stabilise proteins through somewhat different mechanisms. Different mechanisms may be more or less effective for different proteins. Although trehalose has previously shown to possess superior stabilising properties compared to other disaccharides, the acidic nature of the systems presented in this study may be advantageous for the stabilising effect of sucrose.

## 4 Conclusion

This study was conducted to broaden the knowledge regarding the stabilising and anti-aggregating effects trehalose and sucrose have upon the protein HEWL. In particular we investigated how the ability of the protein to form amyloid fibrils was affected by the presence of trehalose or sucrose. Six different systems were compared, Lys/H_2_O, Lys/Tre/H_2_O, and Lys/Suc/H_2_O at both pH 2.0 and pH 3.5, all incubated at *T*_inc_ = 57 °C. Additional sugar-containing systems with an increased *T*_inc_ were also studied (*T*_inc_ = 65 °C and *T*_inc_ = 68 °C for the trehalose and sucrose systems, respectively). It can be concluded that both trehalose and sucrose inhibit the formation of fibrils from HEWL. Partly, by stabilising the native fold of the protein by increasing the thermal stability. However, since the fibrillation was inhibited also at increased *T*_inc_, for both trehalose and sucrose, it is clear that the disaccharides exhibit other positive properties for the inhibition of amyloid fibrils. This is an interesting finding, although not fully understood at this stage, which might be explained by a weakening of hydrogen bonding and hydrophobic interactions between protein monomers due to the presence of the disaccharides, which was previously suggested by Liu *et al.*^52^. Finally, the results indicate that the two disaccharides behave similarly both with respect to stabilising the native state of the protein and to as inhibiting fibril formation. However, at least for the sample conditions in this study, sucrose is seen to be slightly more effective in stabilising the native fold of lysozyme compared to trehalose.

## Author contributions

J. S. and E. K. E. supervised the project. K. A. prepared all samples and performed all measurements. K. A. and F. H. did the AFM imaging. J. A. supported in sample preparation. The first draft of the manuscript was written by K. A., thereafter revised by all authors.

## Conflicts of interest

There are no conflicts to declare.

## Supplementary Material

RA-014-D4RA01171F-s001

RA-014-D4RA01171F-s002

RA-014-D4RA01171F-s003

RA-014-D4RA01171F-s004

RA-014-D4RA01171F-s005

RA-014-D4RA01171F-s006

RA-014-D4RA01171F-s007

RA-014-D4RA01171F-s008

RA-014-D4RA01171F-s009

RA-014-D4RA01171F-s010

RA-014-D4RA01171F-s011

RA-014-D4RA01171F-s012

RA-014-D4RA01171F-s013

RA-014-D4RA01171F-s014

RA-014-D4RA01171F-s015

RA-014-D4RA01171F-s016

RA-014-D4RA01171F-s017

RA-014-D4RA01171F-s018

RA-014-D4RA01171F-s019

RA-014-D4RA01171F-s020
