# Supplementary figures and images for: The inhibition of fibril formation of lysozyme by sucrose and trehalose
Source: RSC Adv. 2024 Apr 15;14(17):11921–31. doi: 10.1039/d4ra01171f (PMC11017192; doi:10.1039/d4ra01171f)

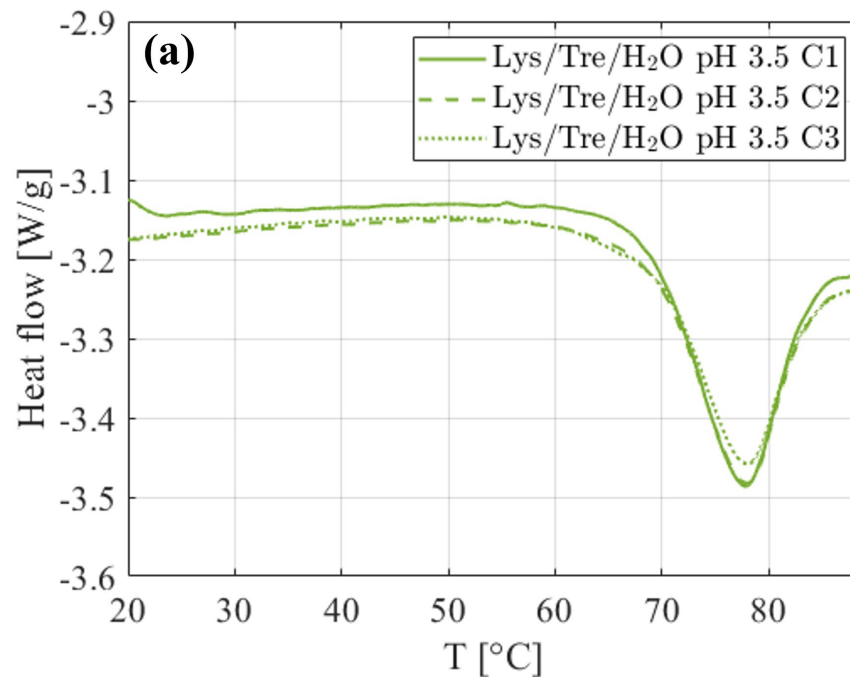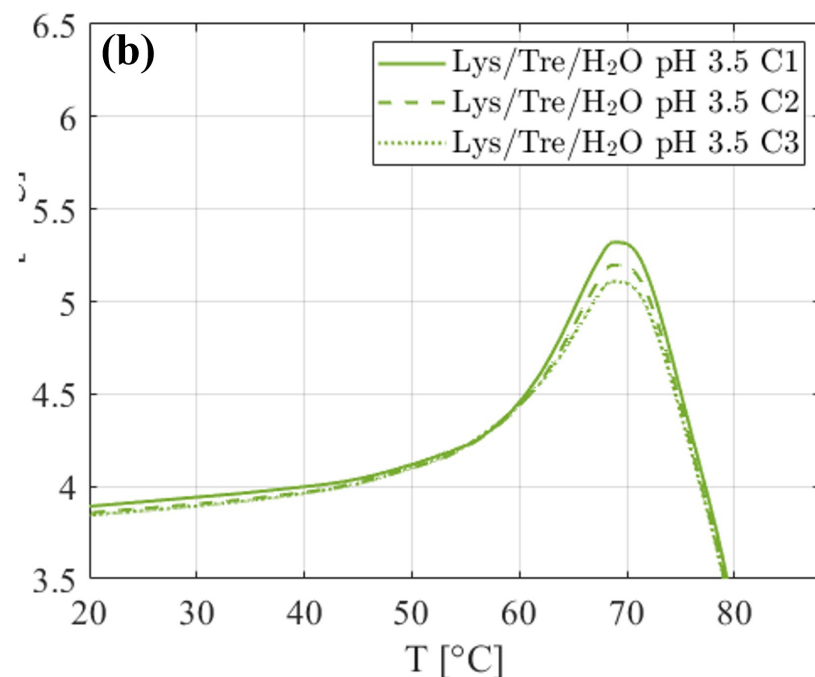

Supplement: RA-014-D4RA01171F-s002 [file RA-014-D4RA01171F-s002.pdf]

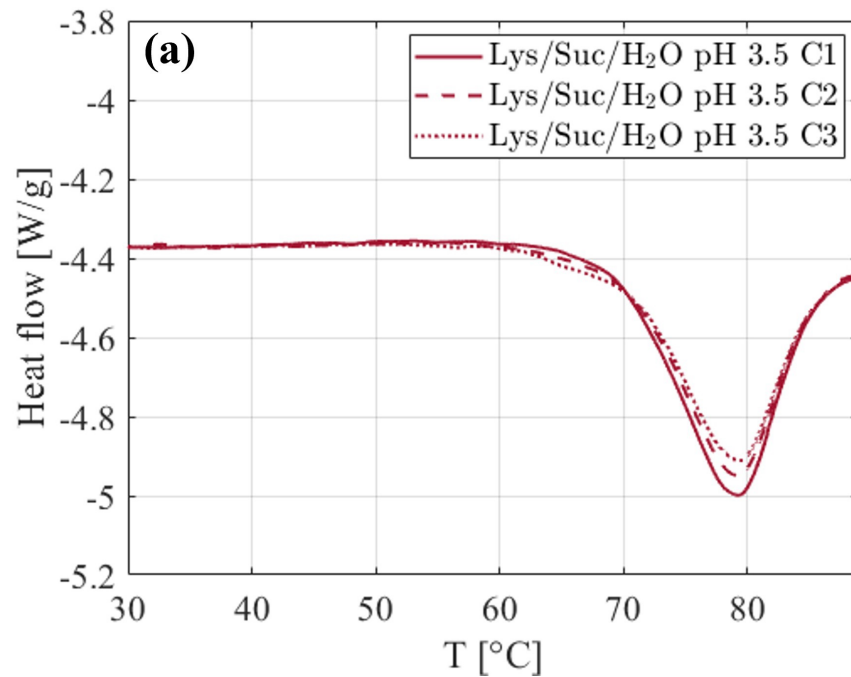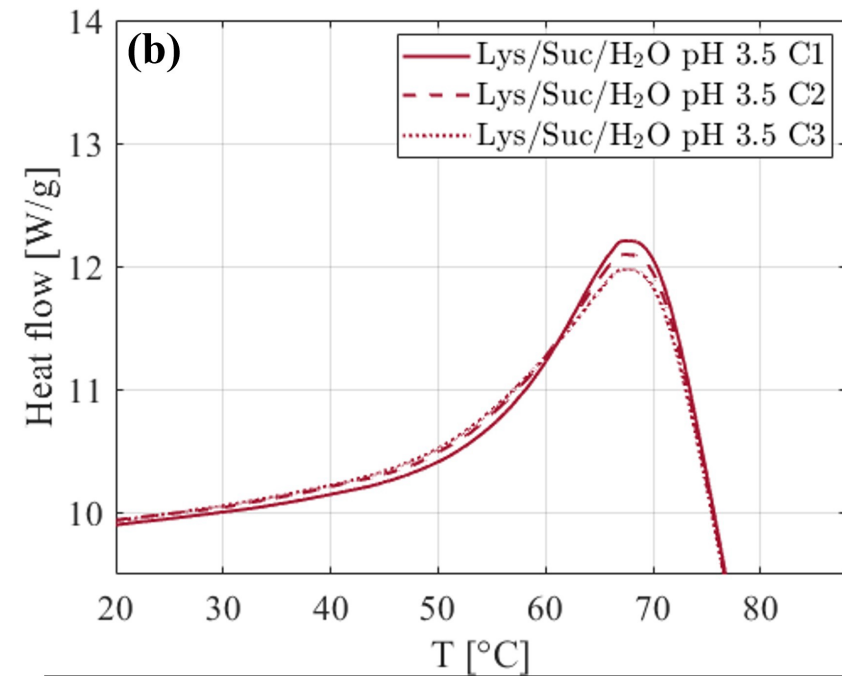

Supplement: RA-014-D4RA01171F-s003 [file RA-014-D4RA01171F-s003.pdf]

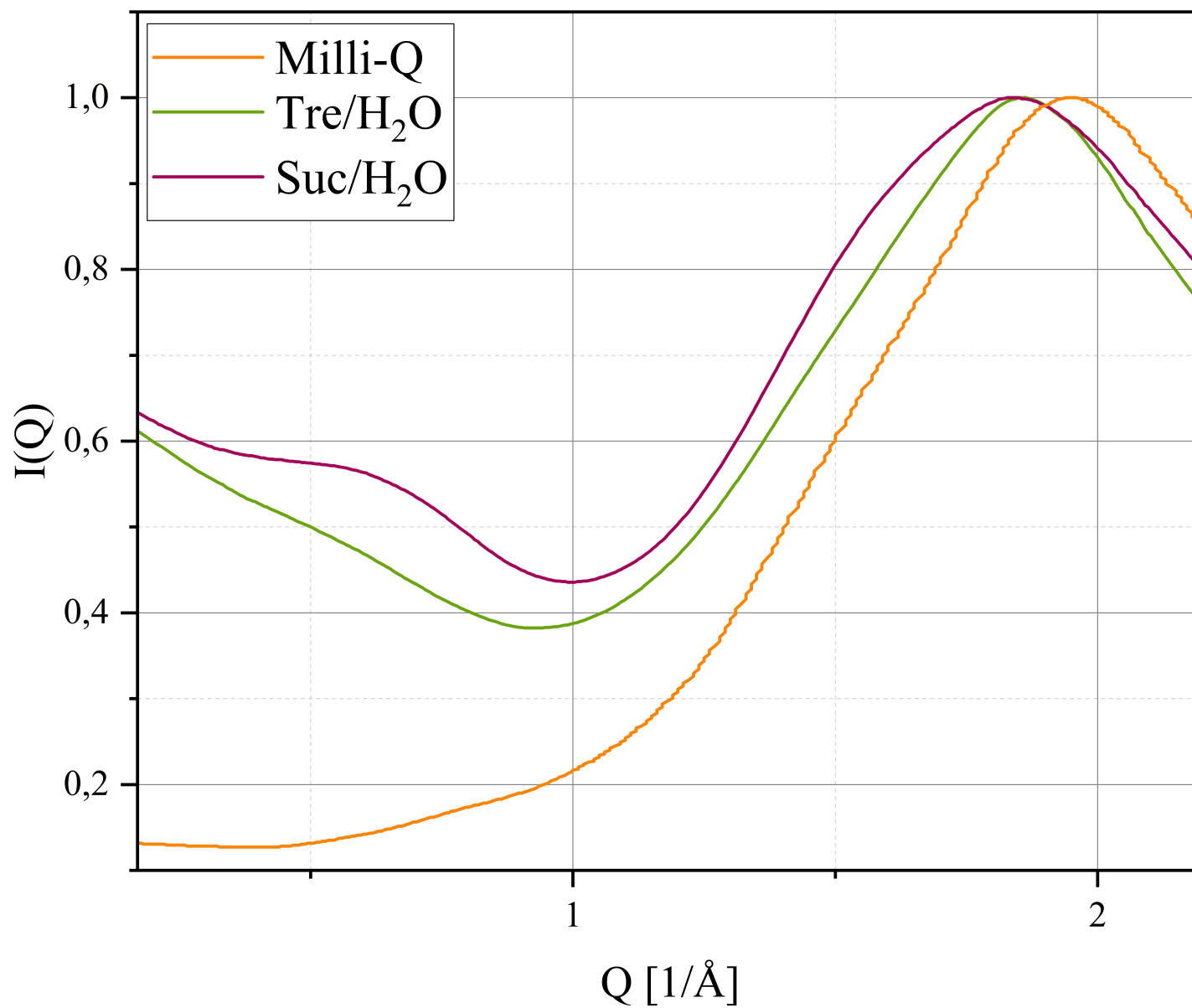

Supplement: RA-014-D4RA01171F-s004 [file RA-014-D4RA01171F-s004.pdf]

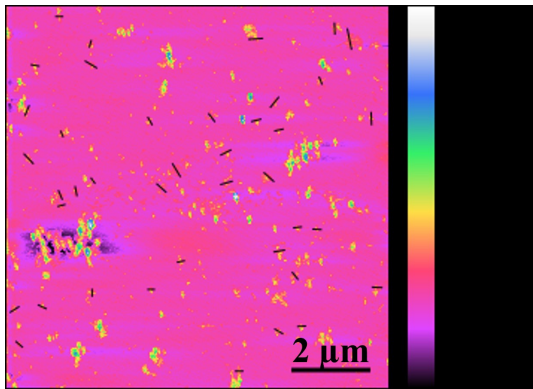

Supplement: RA-014-D4RA01171F-s005 [file RA-014-D4RA01171F-s005.pdf]

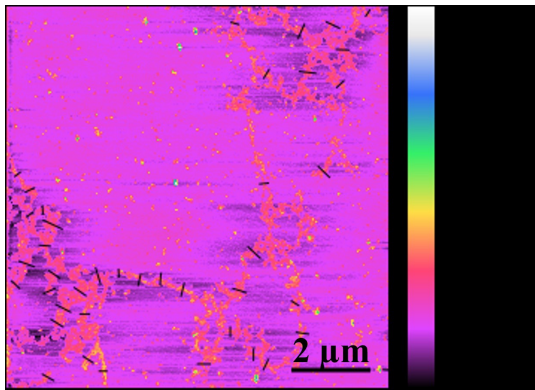

Supplement: RA-014-D4RA01171F-s006 [file RA-014-D4RA01171F-s006.pdf]

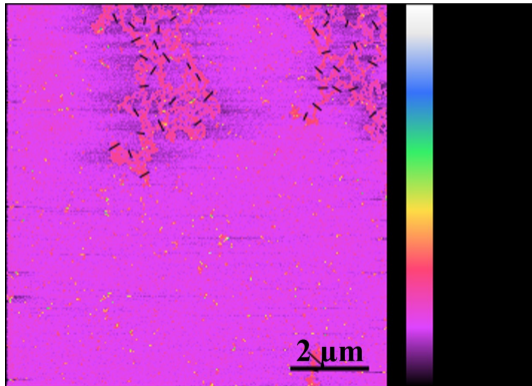

Supplement: RA-014-D4RA01171F-s007 [file RA-014-D4RA01171F-s007.pdf]

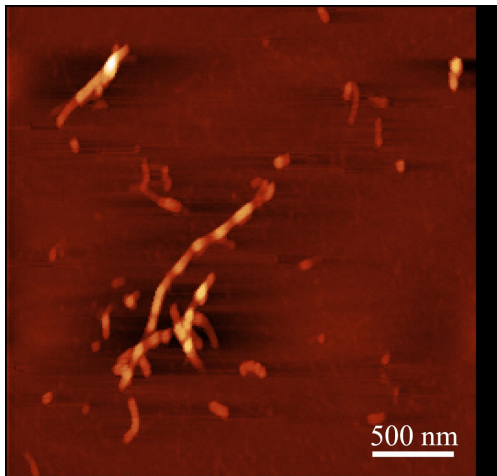

Supplement: RA-014-D4RA01171F-s008 [file RA-014-D4RA01171F-s008.pdf]

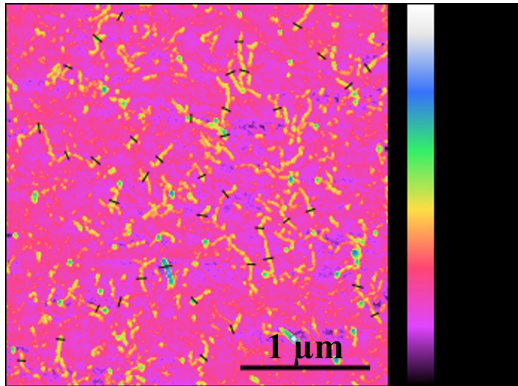

Supplement: RA-014-D4RA01171F-s009 [file RA-014-D4RA01171F-s009.pdf]

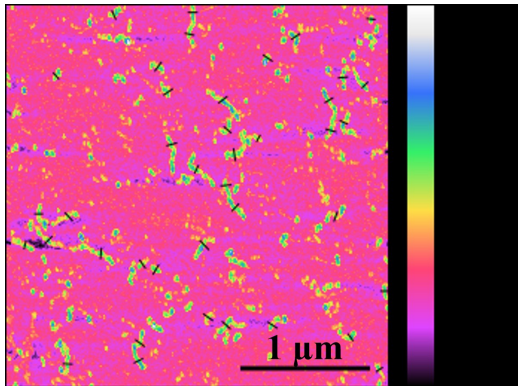

Supplement: RA-014-D4RA01171F-s010 [file RA-014-D4RA01171F-s010.pdf]

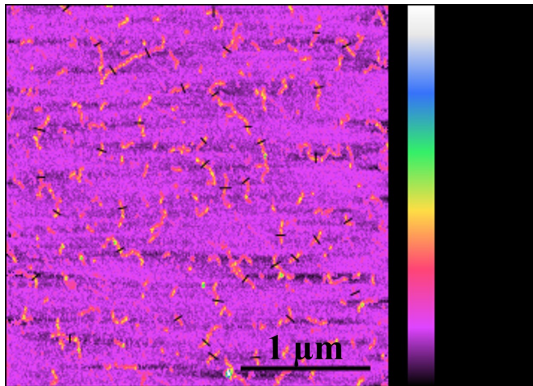

Supplement: RA-014-D4RA01171F-s011 [file RA-014-D4RA01171F-s011.pdf]

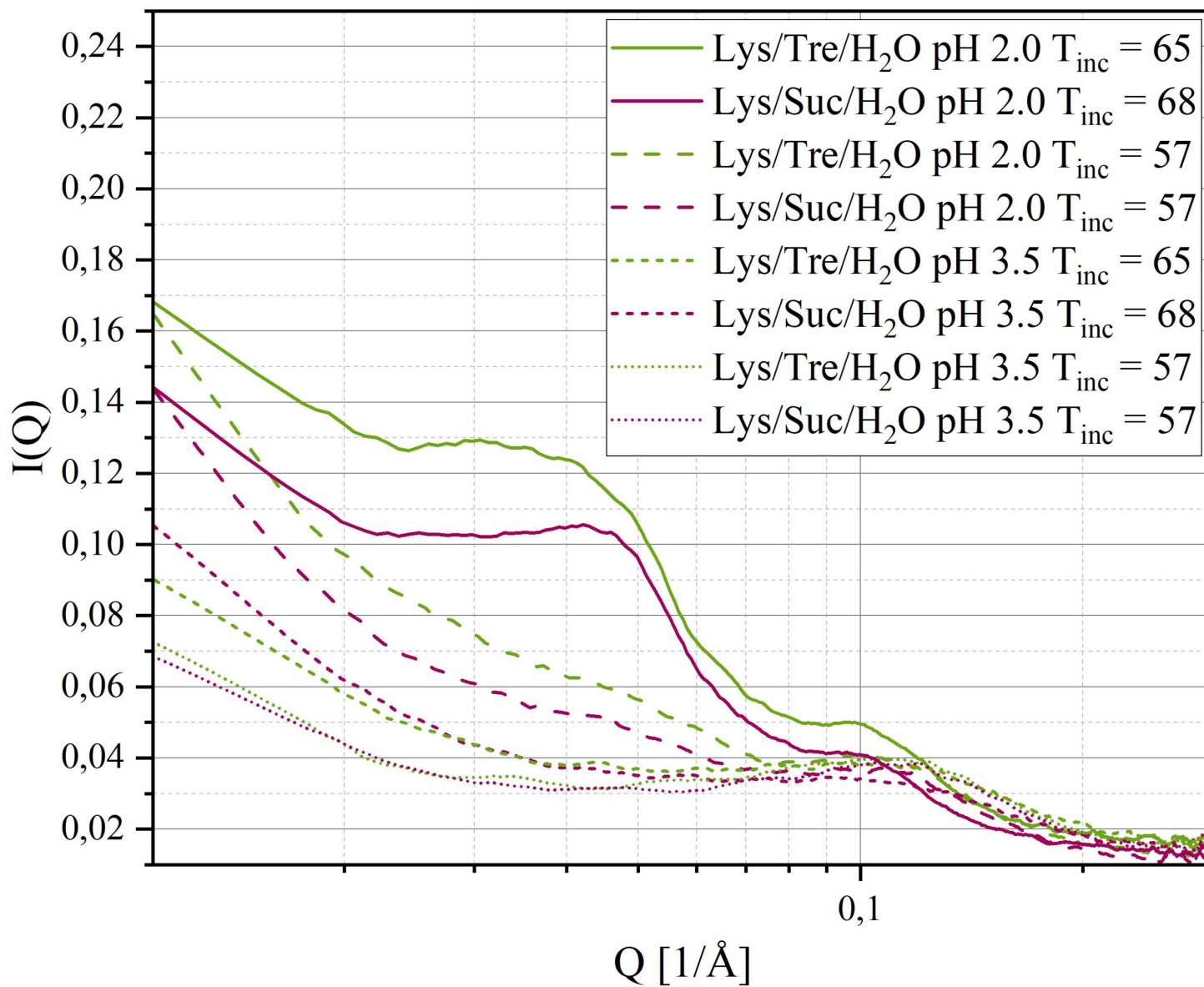

Supplement: RA-014-D4RA01171F-s012 [file RA-014-D4RA01171F-s012.pdf]

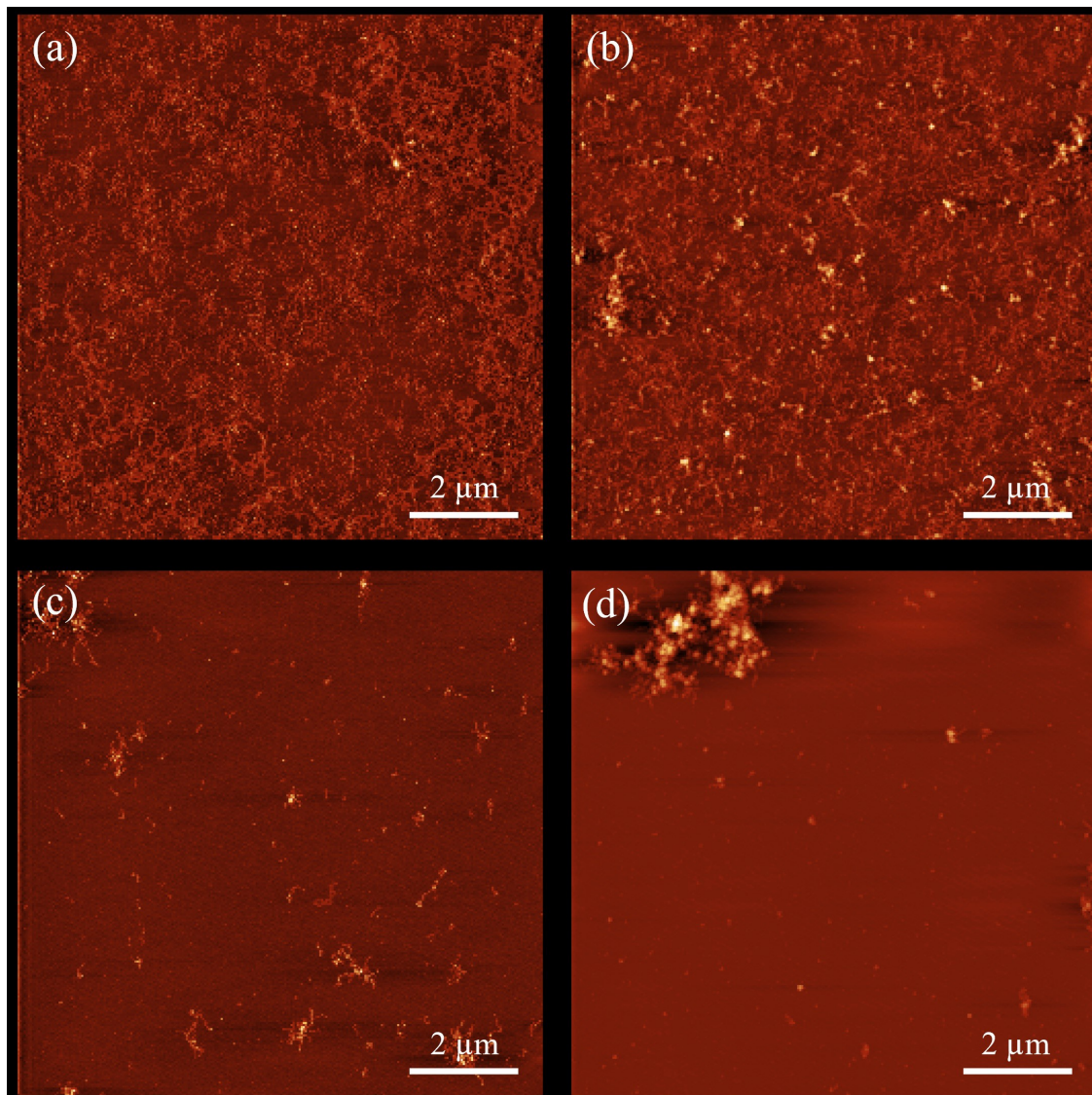

Supplement: RA-014-D4RA01171F-s014 [file RA-014-D4RA01171F-s014.pdf]

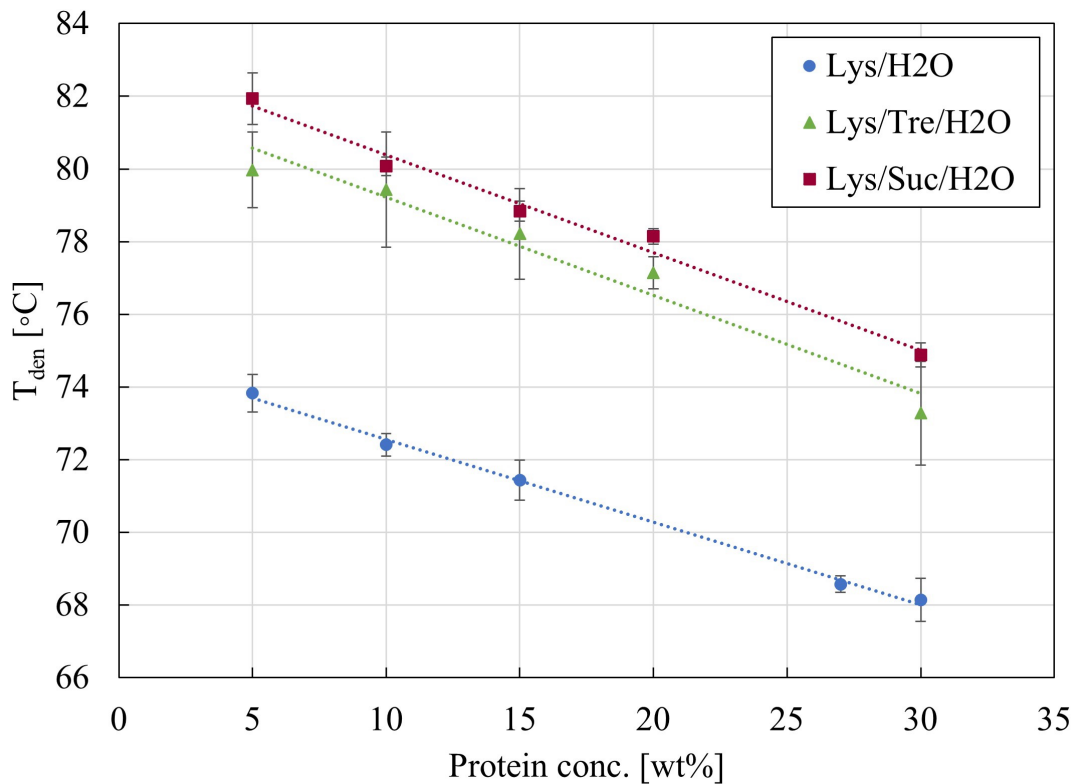

Supplement: RA-014-D4RA01171F-s015 [file RA-014-D4RA01171F-s015.pdf]

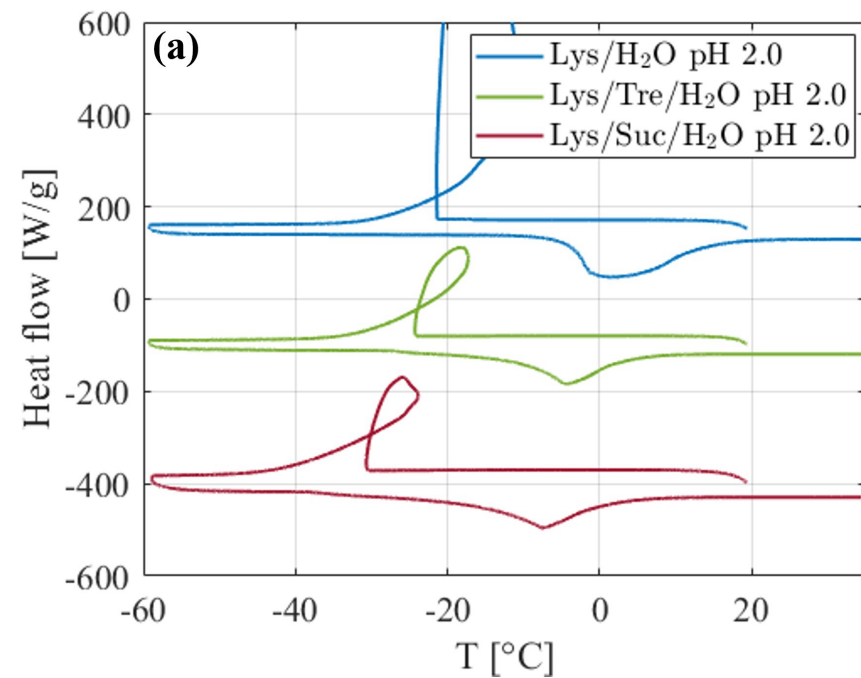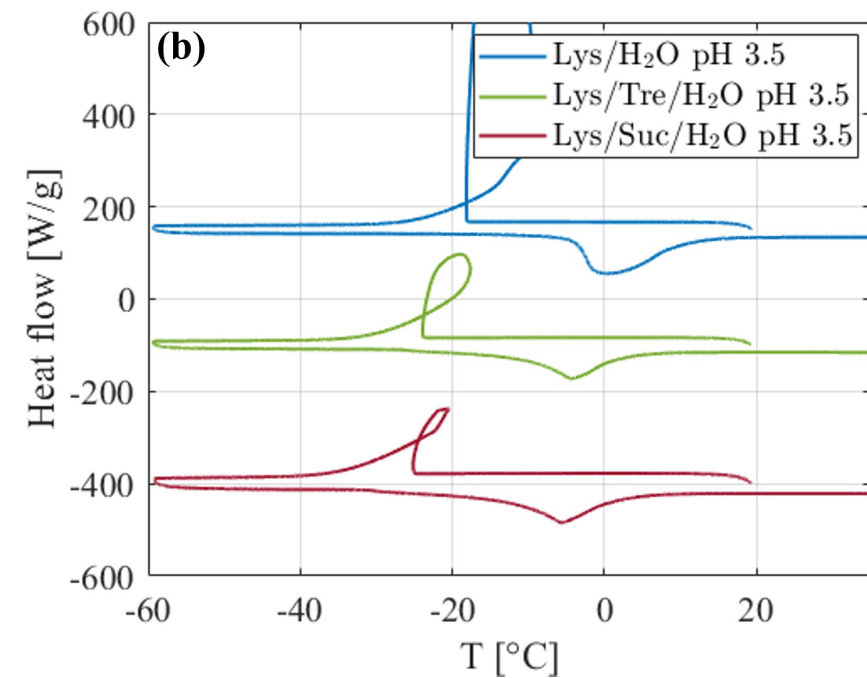

Supplement: RA-014-D4RA01171F-s016 [file RA-014-D4RA01171F-s016.pdf]

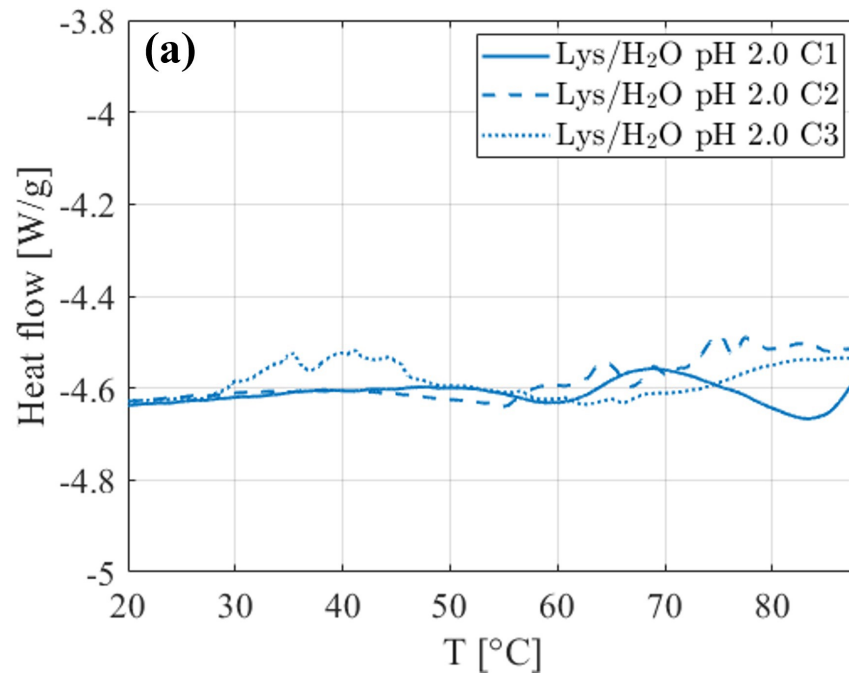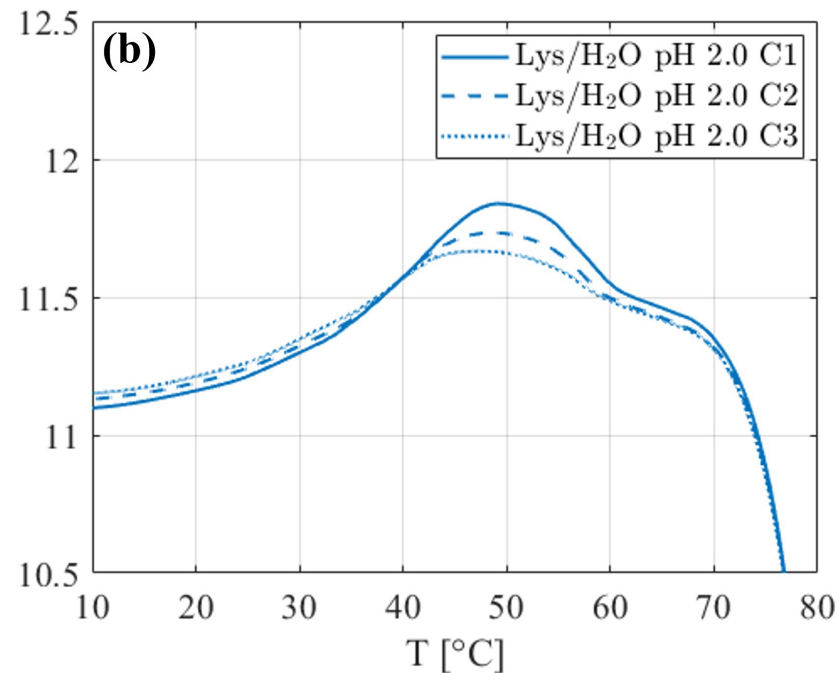

Supplement: RA-014-D4RA01171F-s017 [file RA-014-D4RA01171F-s017.pdf]

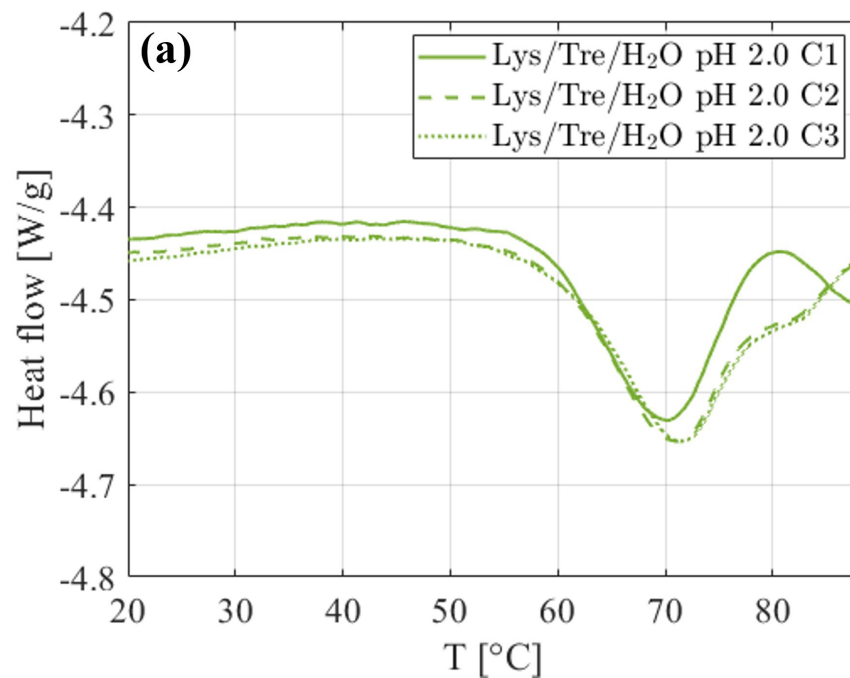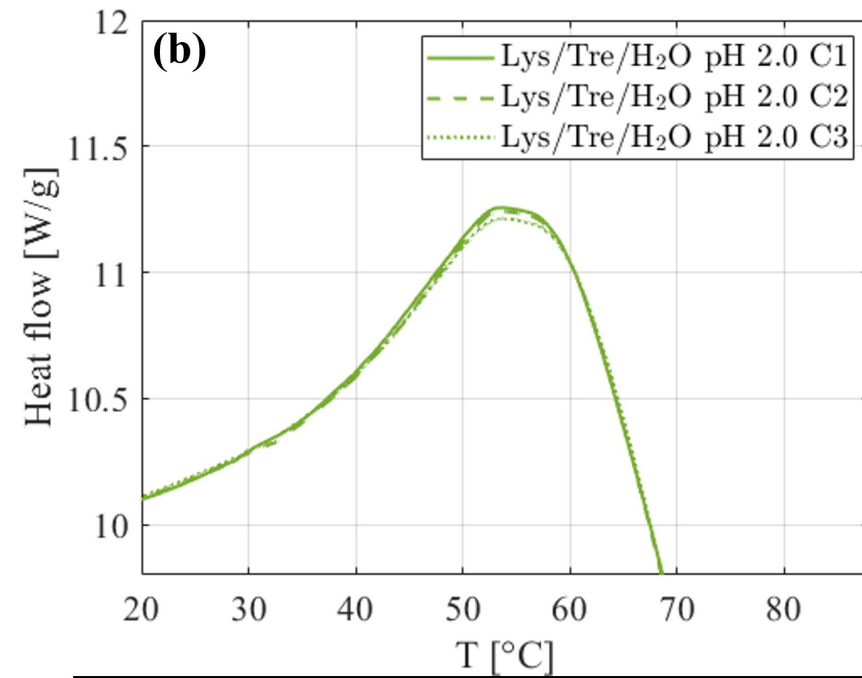

Supplement: RA-014-D4RA01171F-s018 [file RA-014-D4RA01171F-s018.pdf]

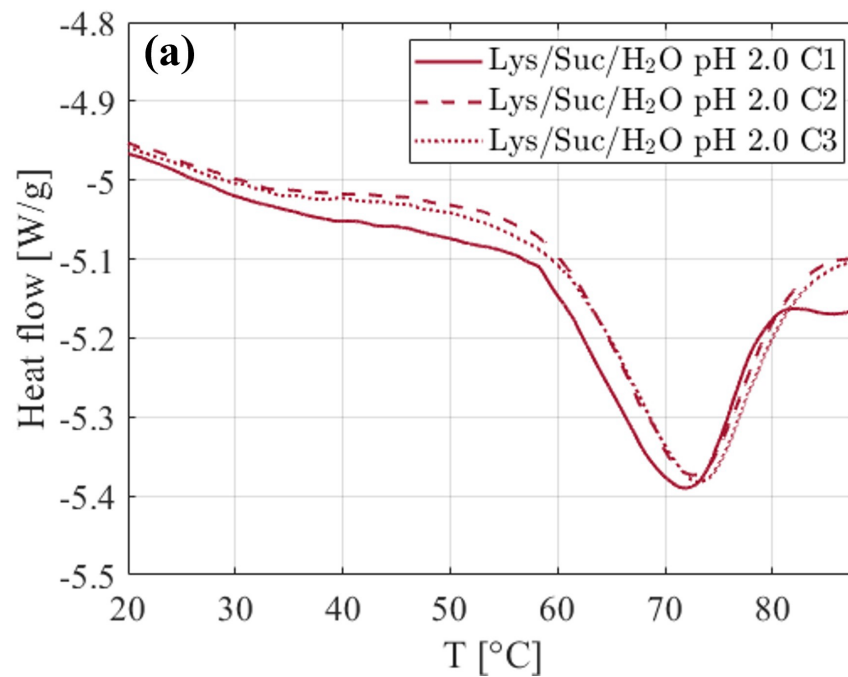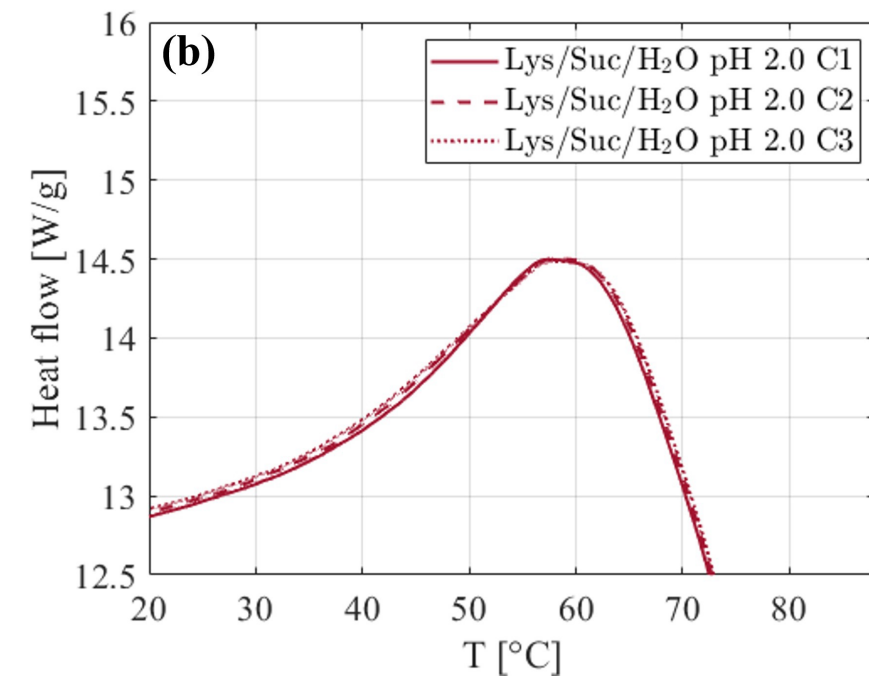

Supplement: RA-014-D4RA01171F-s019 [file RA-014-D4RA01171F-s019.pdf]

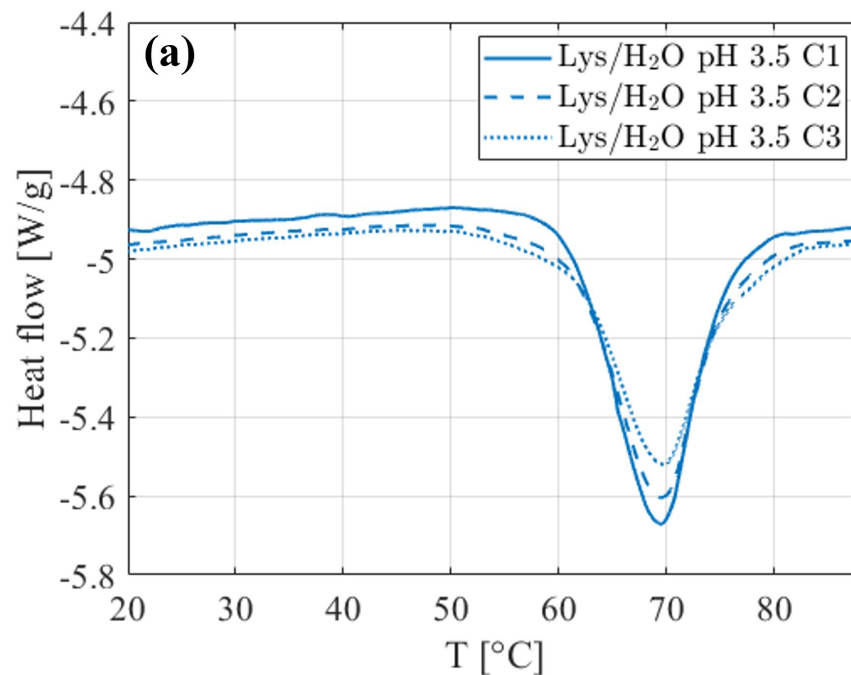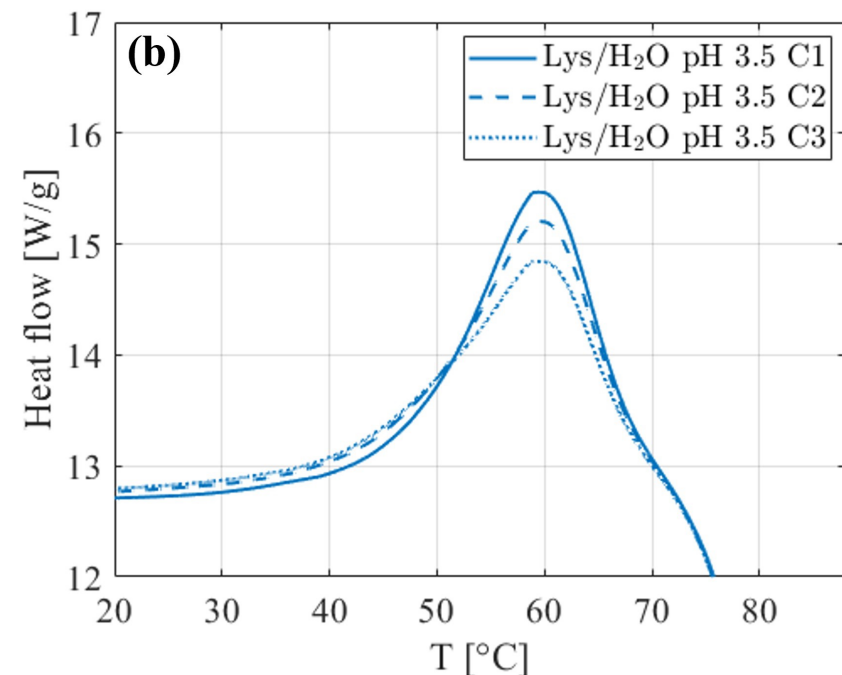

Supplement: RA-014-D4RA01171F-s020 [file RA-014-D4RA01171F-s020.pdf]
