# Supplementary material for: The inhibition of fibril formation of lysozyme by sucrose and trehalose
Source: RSC Adv. 2024 Apr 15;14(17):11921–31. doi: 10.1039/d4ra01171f (PMC11017192; doi:10.1039/d4ra01171f)
Supplement: RA-014-D4RA01171F-s013 [file RA-014-D4RA01171F-s013.pdf]

Electronic Supplementary Material (ESI) for RSC Advances.
This journal is © The Royal Society of Chemistry 2024

Electronic Supplementary Information for "The Inhibition of Fibril Formation of Lysozyme by Sucrose and Trehalose"

Kajsa Ahlgren, [a,*] Fritjof Havemeister, [b] Julia Andersson,[a] Elin K. Esbjörner, [b] Jan Swenson[a]

[a] *Division of Nano- Biophysics, Department of Physics, Chalmers University of Technology, 412 96 Gothenburg, Sweden.*
[b] *Division of Chemical Biology, Department of Life Sciences, Chalmers University of Technology, SE 412 96, Gothenburg, Sweden.*
[*] *Corresponding author: kajsa.ahlgren@chalmers.se*

Contents

1 X-ray scattering **2**

2 Atomic Force Microscopy **3**

3 Differential Scanning Calorimetry **7**

1 X-ray scattering

Figure S1 shows the un-normalised SAXS data for the Lys/Tre/H_2O and Lys/Suc/H_2O systems at pH 2.0 and 3.5. The comparison is based on different T_{inc} and shows that the Q_2-peak is shifted towards slightly lower Q-values once the T_{inc} is increased for the system. This implies that the distance separating the protein molecules increase when the T_{inc} is closer to the denaturation point of respective system. This is valid for both the trehalose as well as the sucrose system.

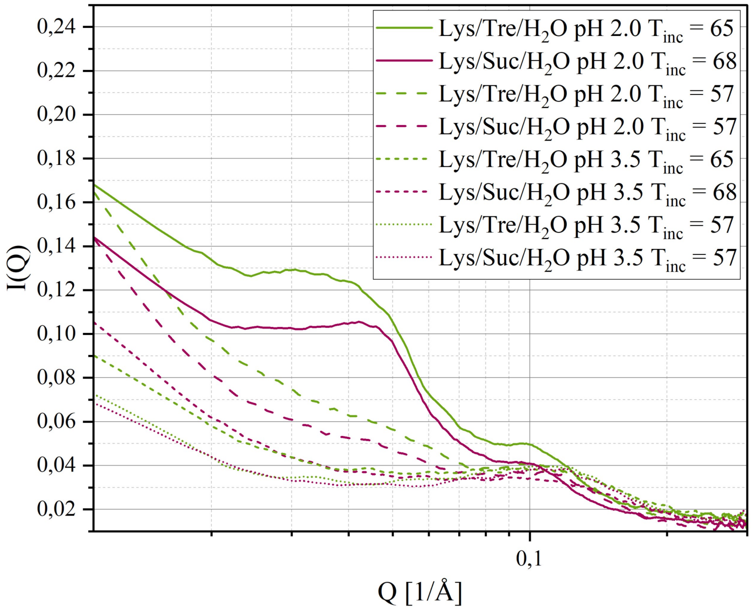

Figure S1 A comparison of the SAXS peaks expressed for the Lys/Tre/H_2O and Lys/Suc/H_2O systems at different pH and incubation temperatures

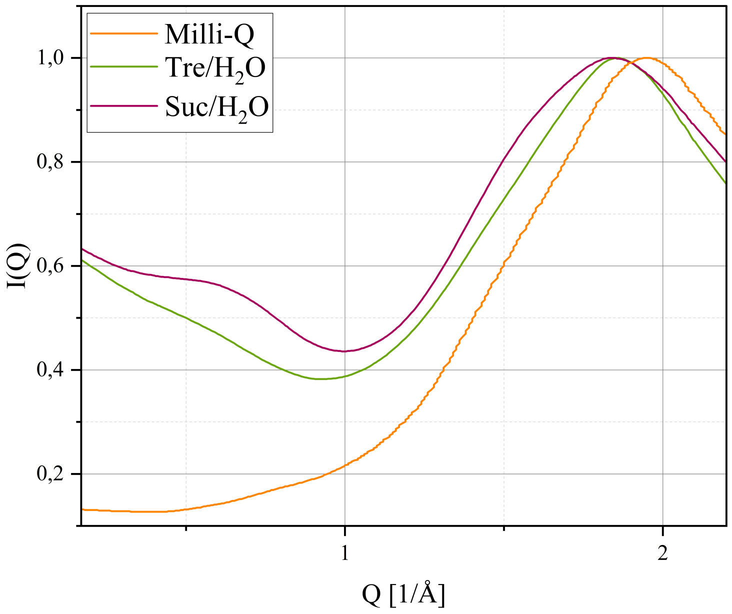

Figure S2 WAXS data of Tre/H_2O and Suc/H_2O solutions compared to pure Milli-Q-water. The sugar:water ratio was same in these systems as in the three-component systems.

2 Atomic Force Microscopy

Figures S3-5 and S7-9 shows the AFM scans with a colour map to analyse the height of the protein aggregates. The small black lines are the point at which the heights were measured.

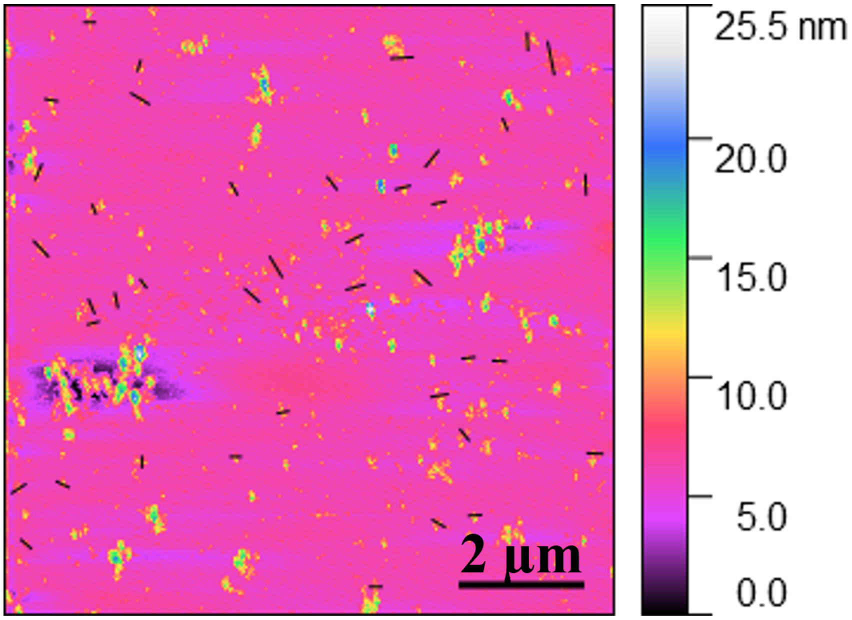

Figure S3 AFM image of Lys/H_2O at pH 3.5 including colour map.

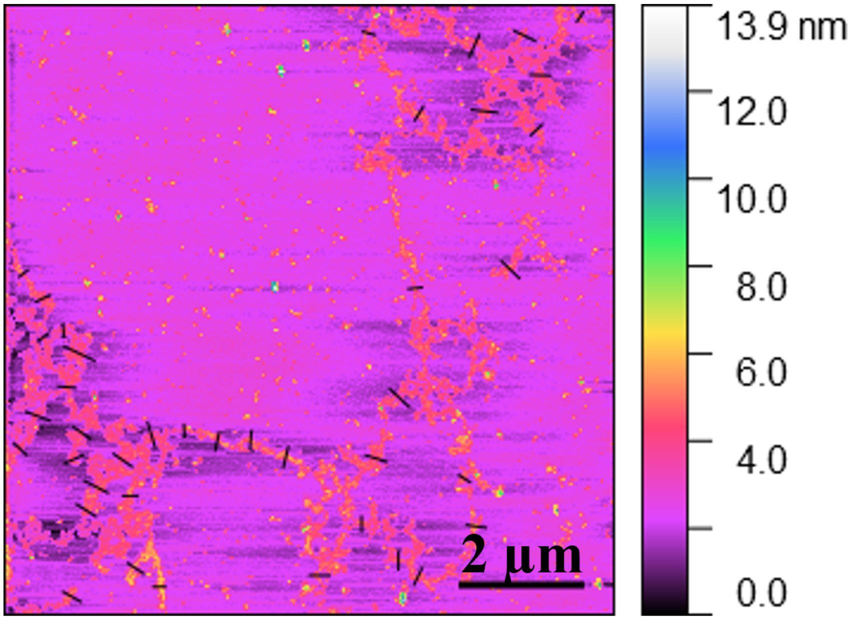

Figure S4 AFM image of Lys/Tre/H_2O at pH 3.5 including colour map.

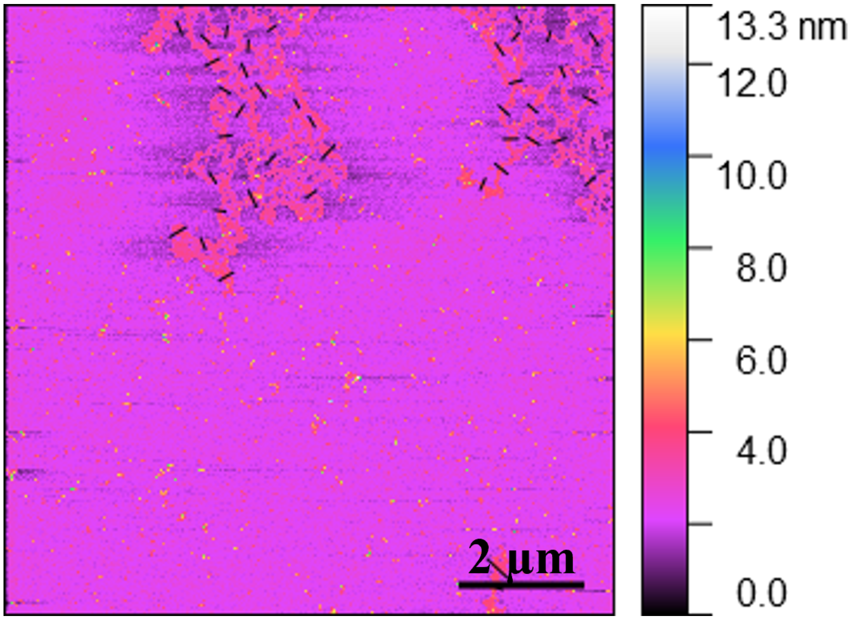

Figure S5 AFM image of Lys/Suc/H_2O at pH 3.5 including colour map.

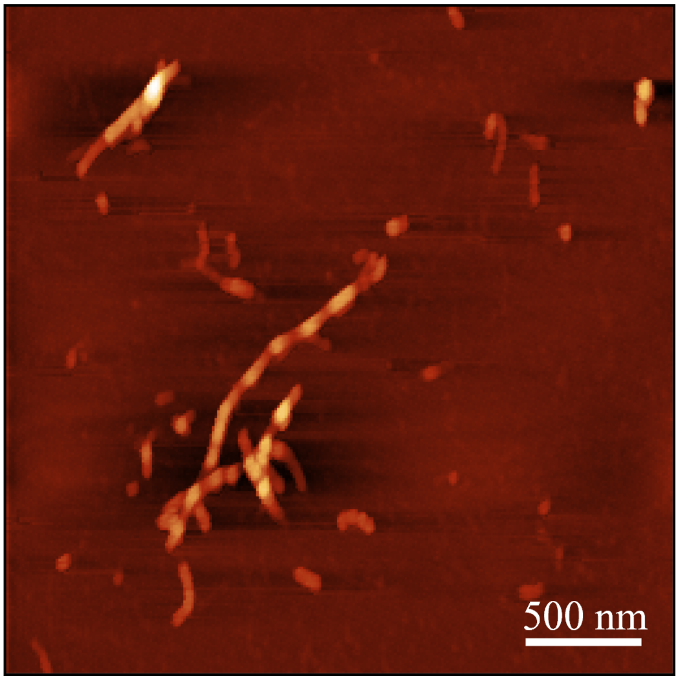

Figure S6 AFM image of Lys/H_2O at pH 2.0.

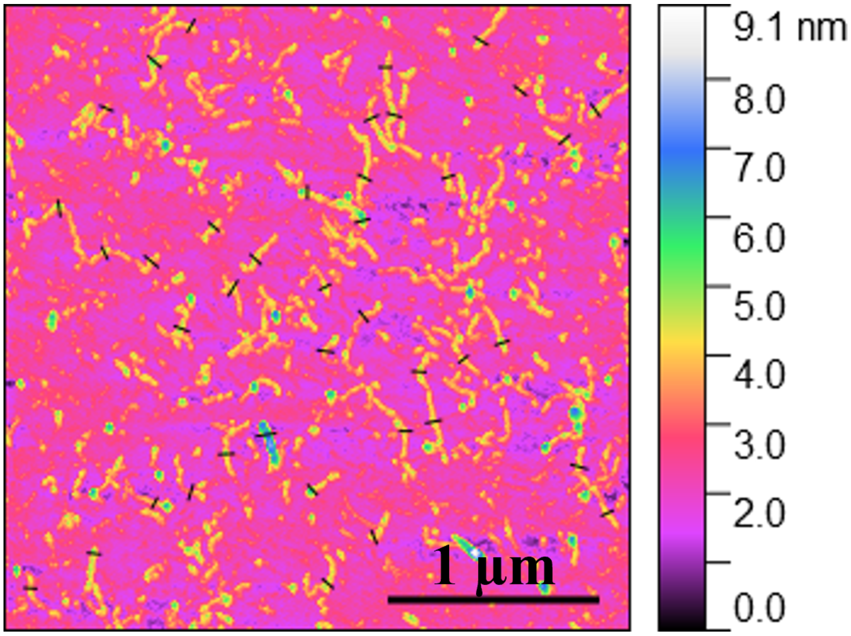

Figure S7 AFM image of Lys/H_2O at pH 2.0 including colour map.

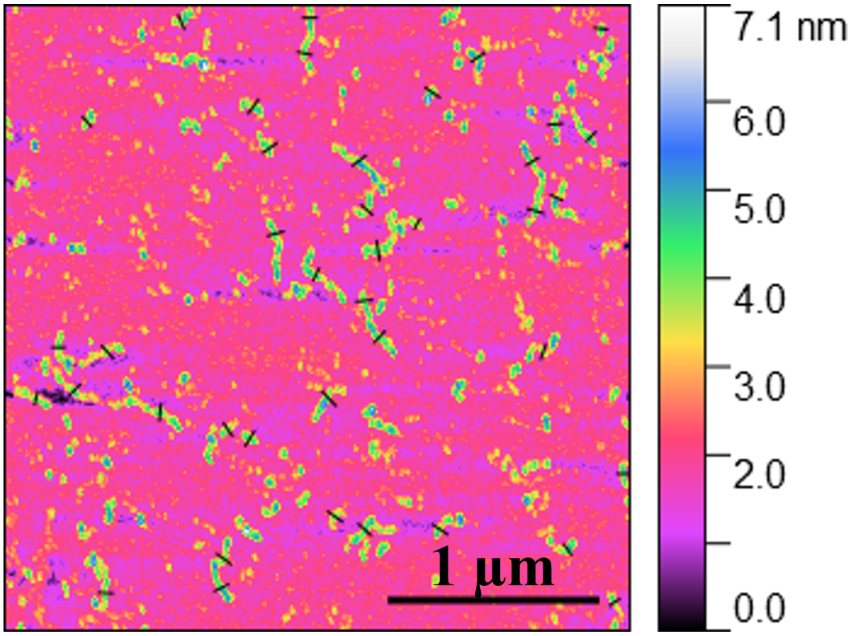

Figure S8 AFM image of Lys/Tre/H_2O at pH 2.0 including colour map.

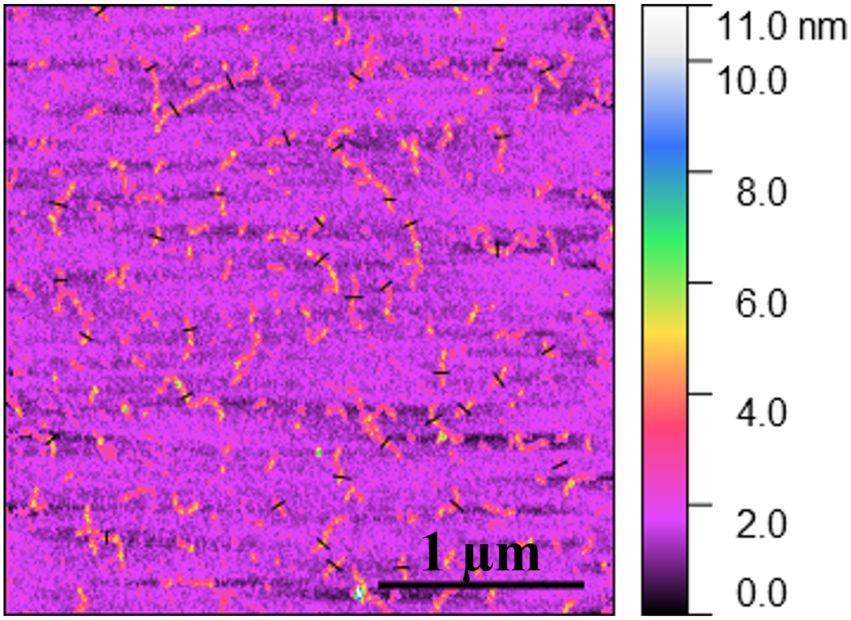

Figure S9 AFM image of Lys/Suc/H_2O at pH 2.0 including colour map.

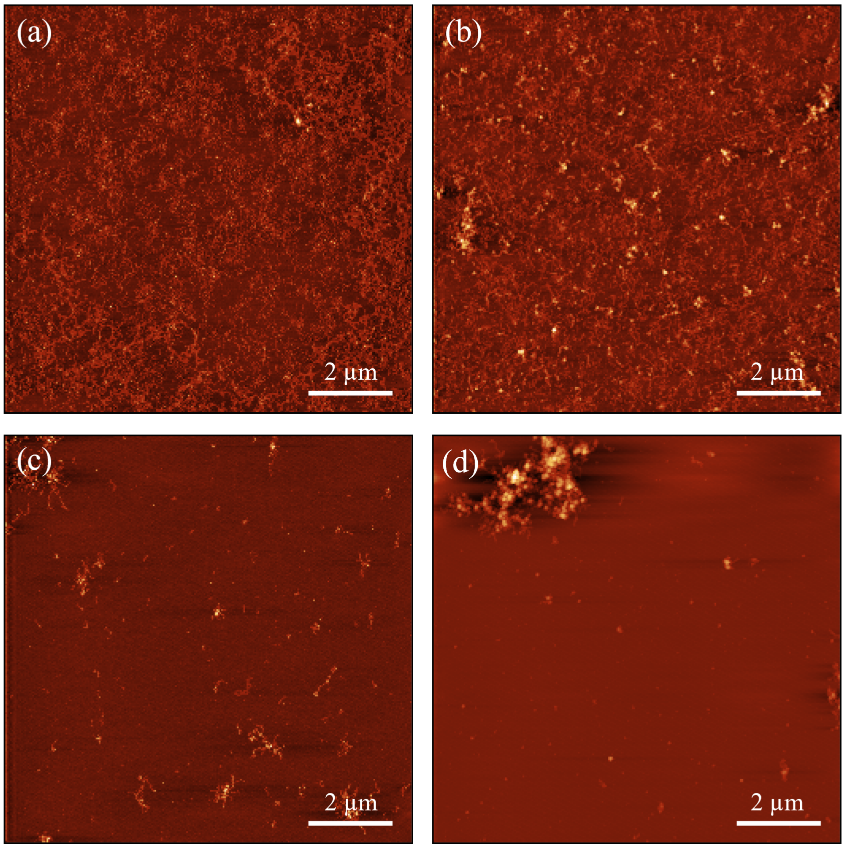

Figure S10 Representative AFM images of (a) Lys/Suc/H_2O and (b) Lys/Tre/H_2Oat pH 2.0 , and (c) Lys/Suc/H_2O and (d) Lys/Tre/H_2O at pH 3.5. The sucrose containing systems have been incubated at 68 °C and the trehalose systems at 65°C.

3 Differential Scanning Calorimetry

Figure S12(a) shows representative DSC curves over the crystallisation and melting process for the Lys/H_2O, Lys/Tre/H_2O, and Lys/Suc/H_2O systems at pH 2.0. Figure S12(b) shows the curves for the respective systems at pH 3.5. The crystallisation process is seen as a dramatic exotherm peak during the cooling cycle and the melting process is seen as a endotherm peak during the heating cycle.

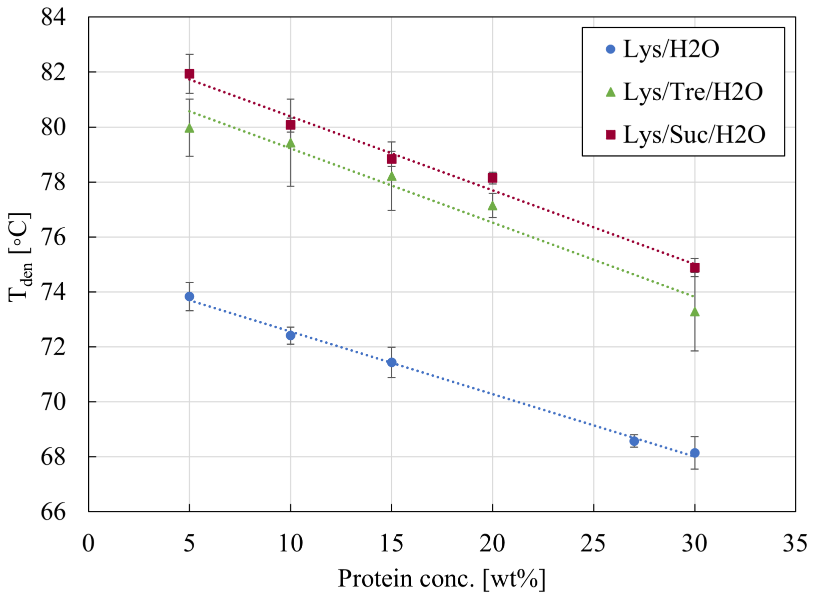

Figure S11 Denaturation temperatures for the Lys/H_2O, Lys/Tre/H_2O, and Lys/Suc/H_2O systems without any prior incubation or altering of pH as a function of the protein concentration. The sugar:water weight ratio was kept constant in all three-component systems. The measurements were performed at a heating rate of 2 °C/min.

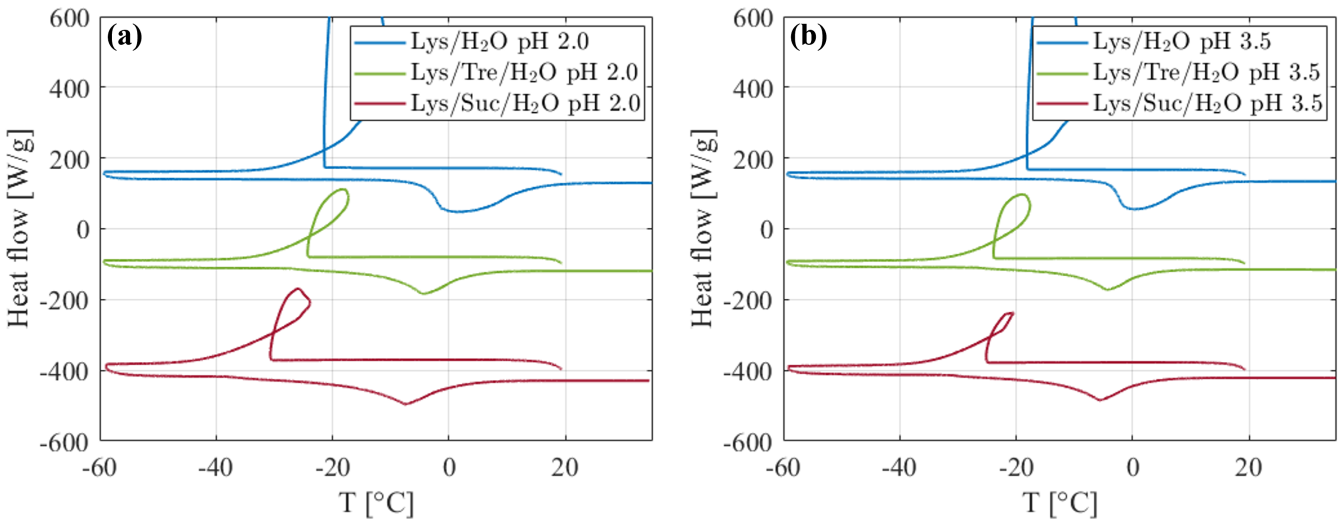

Figure S12 Representative DSC curves for the Lys/H_2O, Lys/Tre/H_2O, and Lys/Suc/H_2O systems at pH 2.0 (a) and pH 3.5 (b) of the crystallisation event measured at a cooling rate of 10 °C/min. The curves have been vertically shifted for clarity.

Figures S13 - S15 displays the unfolding and folding, respectively, of the Lys/H_2O, Lys/Tre/H_2O, and Lys/Suc/H_2O systems at pH 2.0 during three cycles. In Figure S13(a) it can be seen that the protein does not unfold during the second and third cycle and in Figure S13(b) that the folding of the protein also decrease for each cycle. This is not observed to the same extent for the sugar containing systems in Figures S14 and S15 and the protein denaturation is seen to be more or less reversible. In addition, Figures S16 - S18 show the unfolding and folding, respectively, of Lys/H_2O, Lys/Tre/H_2O, and Lys/Suc/H_2O at pH 3.5 during three cycles. The protein denaturation is not fully reversible, due to protein aggregation, for any of the systems at pH 3.5. However, the denaturation can be seen to be slightly more reversible in the presence of the disaccharides.

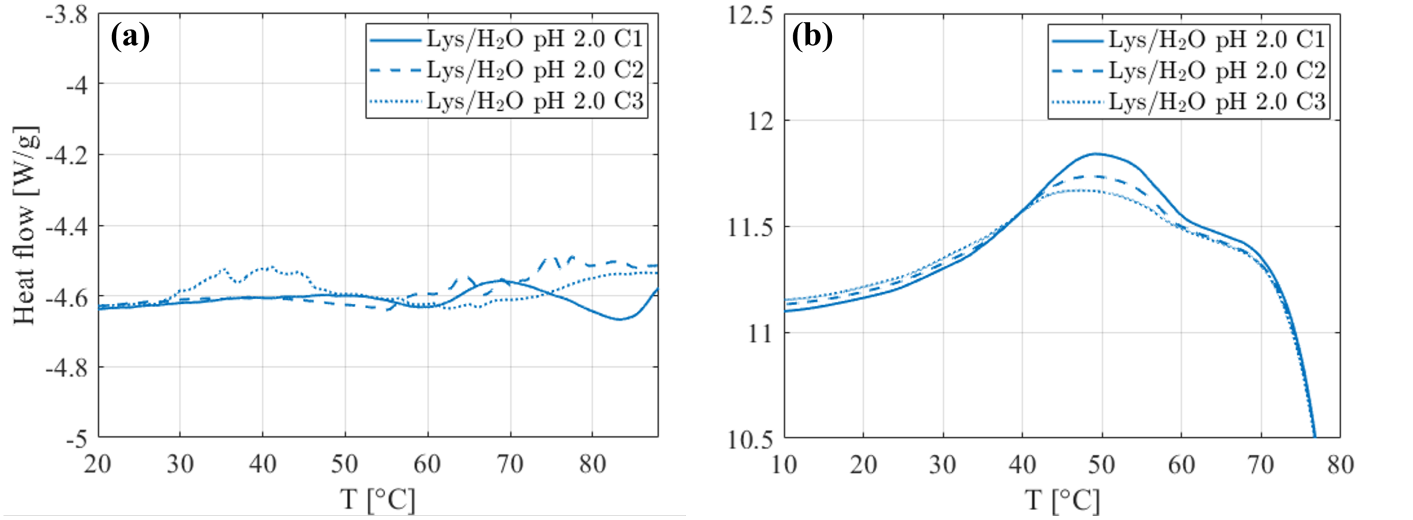

Figure S13 Representative DSC curves for the unfolding (a) and folding (b) of the Lys/H_2O systems at pH 2.0.

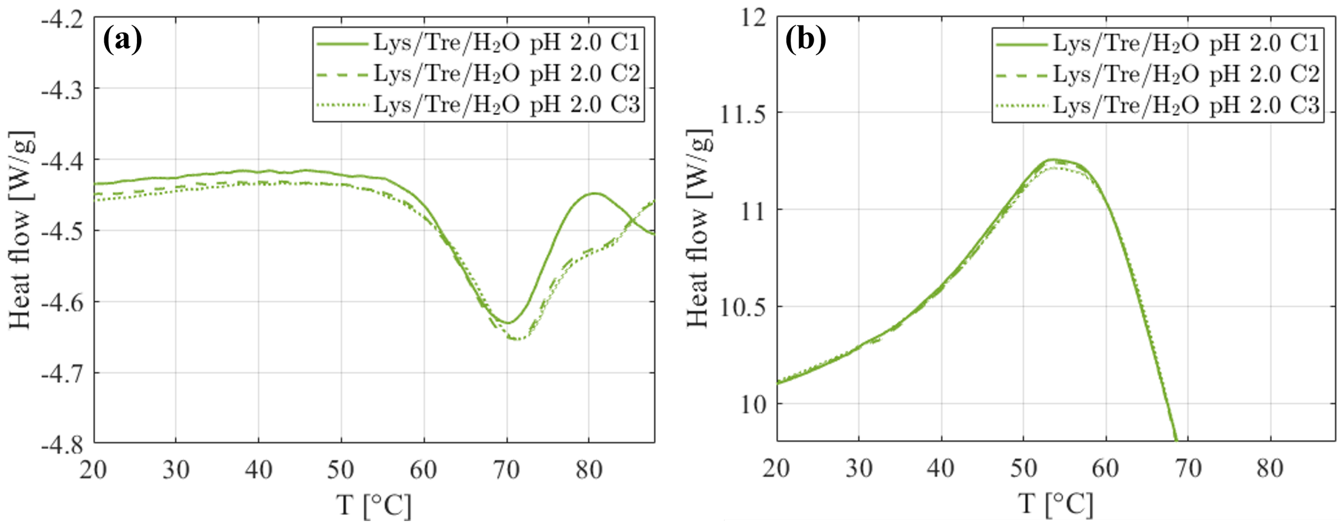

Figure S14 Representative DSC curves for the unfolding (a) and folding (b) of the Lys/Tre/H_2O systems at pH 2.0.

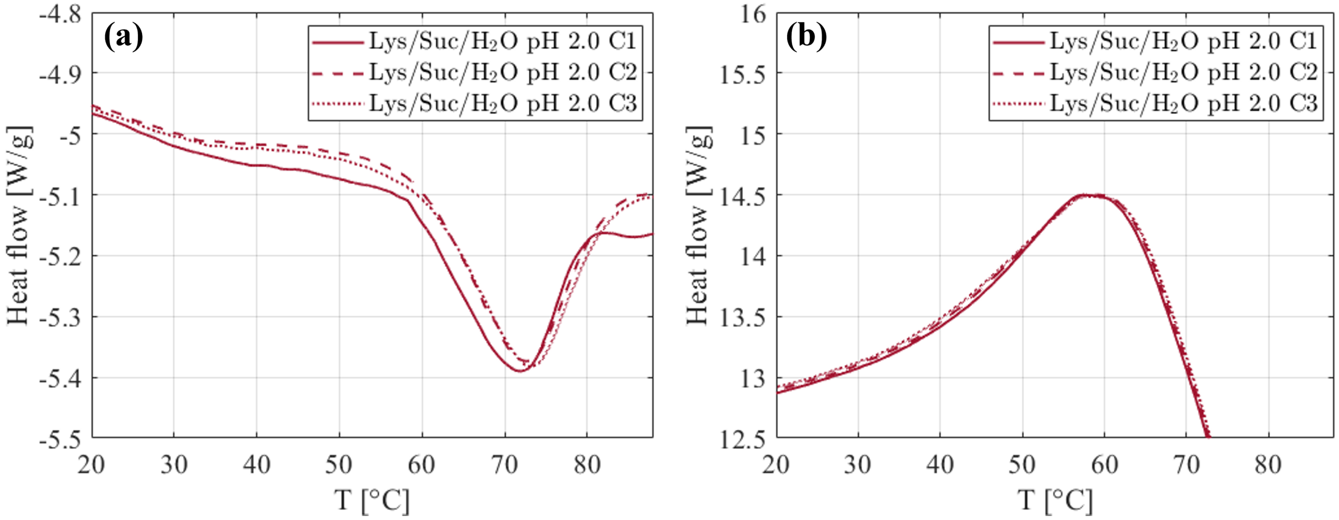

Figure S15 Representative DSC curves for the unfolding (a) and folding (b) of the Lys/Suc/H_2O systems at pH 2.0.

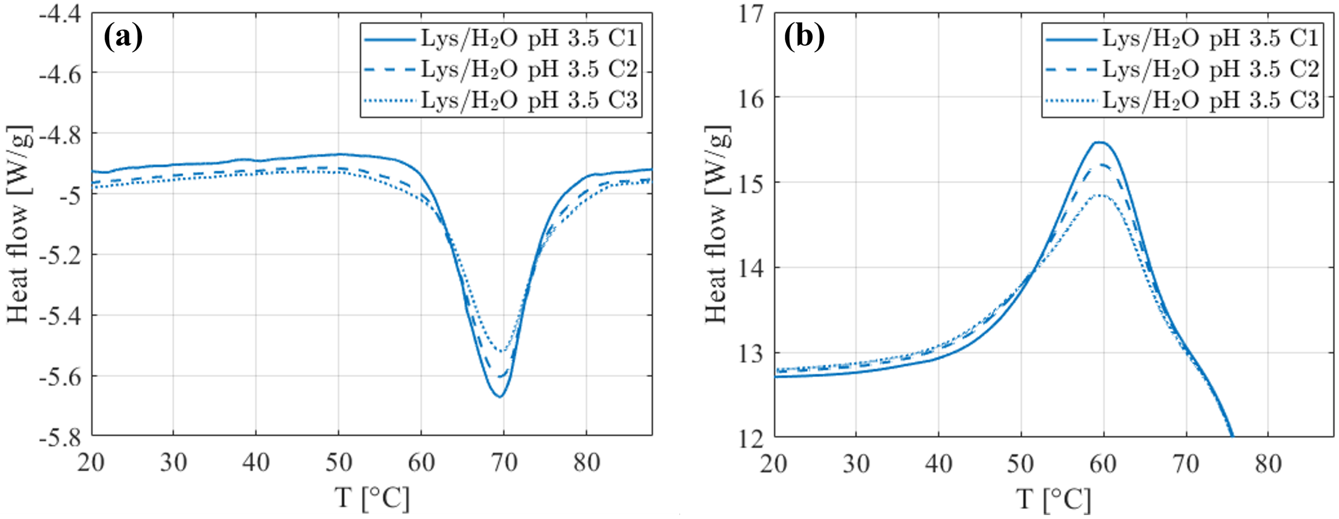

Figure S16 Representative DSC curves for the unfolding (a) and folding (b) of the Lys/H_2O systems at pH 3.5.

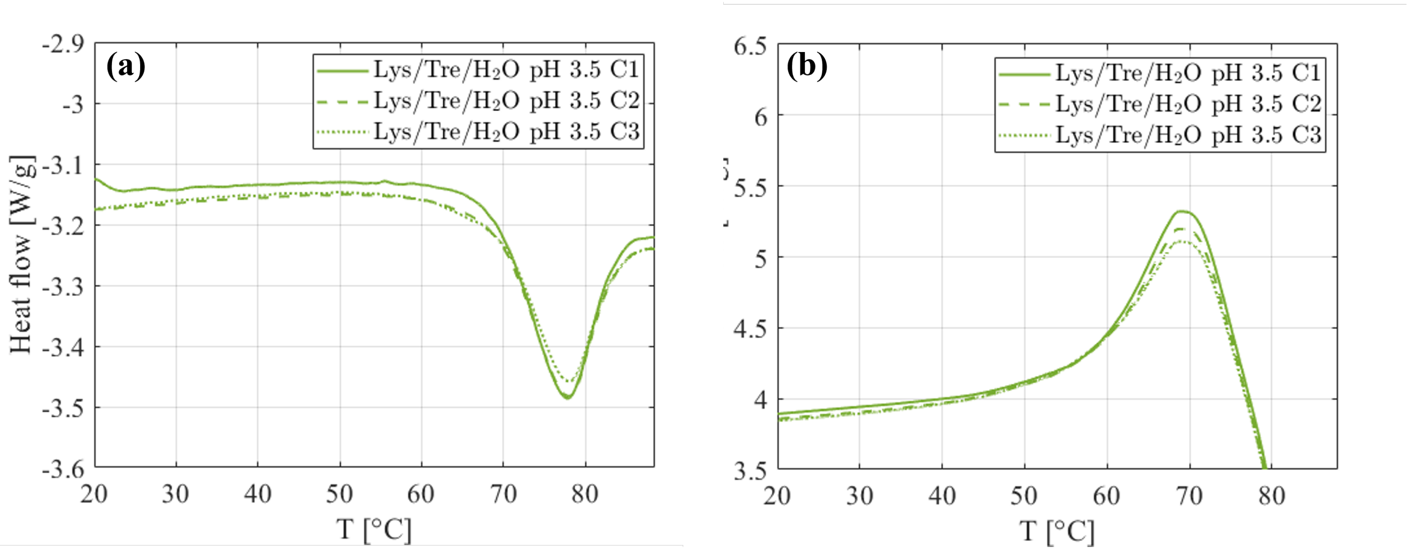

Figure S17 Representative DSC curves for the unfolding (a) and folding (b) of the Lys/Tre/H_2O systems at pH 3.5.

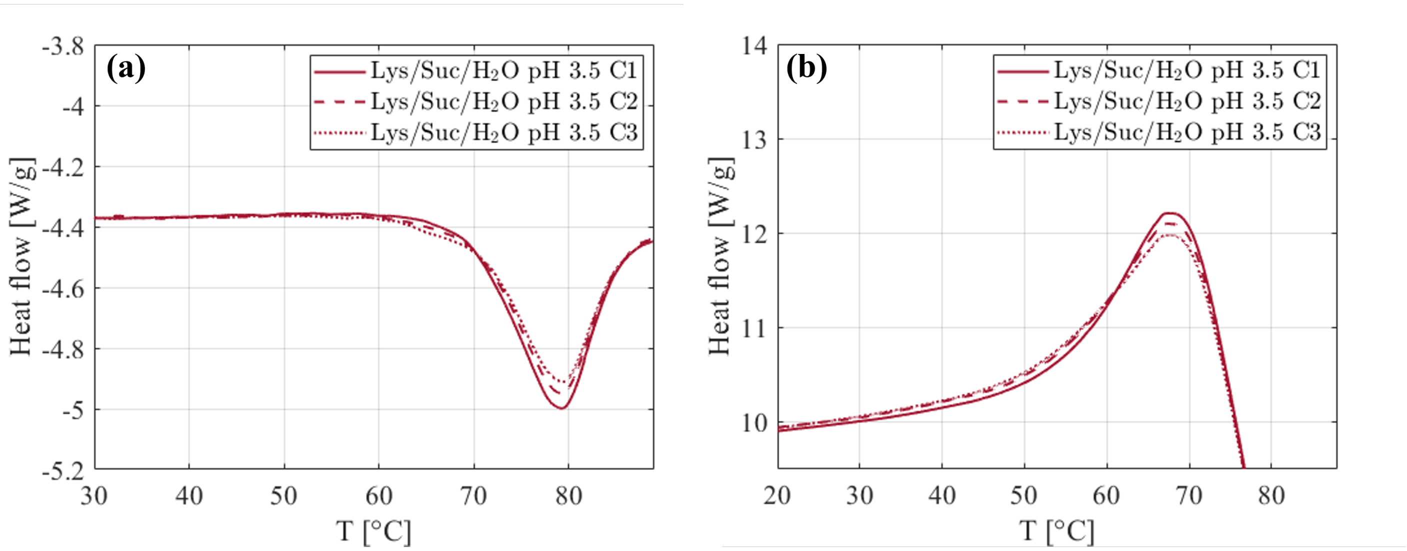

Figure S18 Representative DSC curves for the unfolding (a) and folding (b) of the Lys/Suc/H_2O systems at pH 3.5.
